# Supplementary material for: IGF2BP1 phosphorylation in the disordered linkers regulates ribonucleoprotein condensate formation and RNA metabolism
Source: Nat Commun. 2024 Oct 20;15:9054. doi: 10.1038/s41467-024-53400-4 (PMC11490574; doi:10.1038/s41467-024-53400-4)
Supplement: Supplementary file 1 — Supplementary Information [file 41467_2024_53400_MOESM1_ESM.pdf]

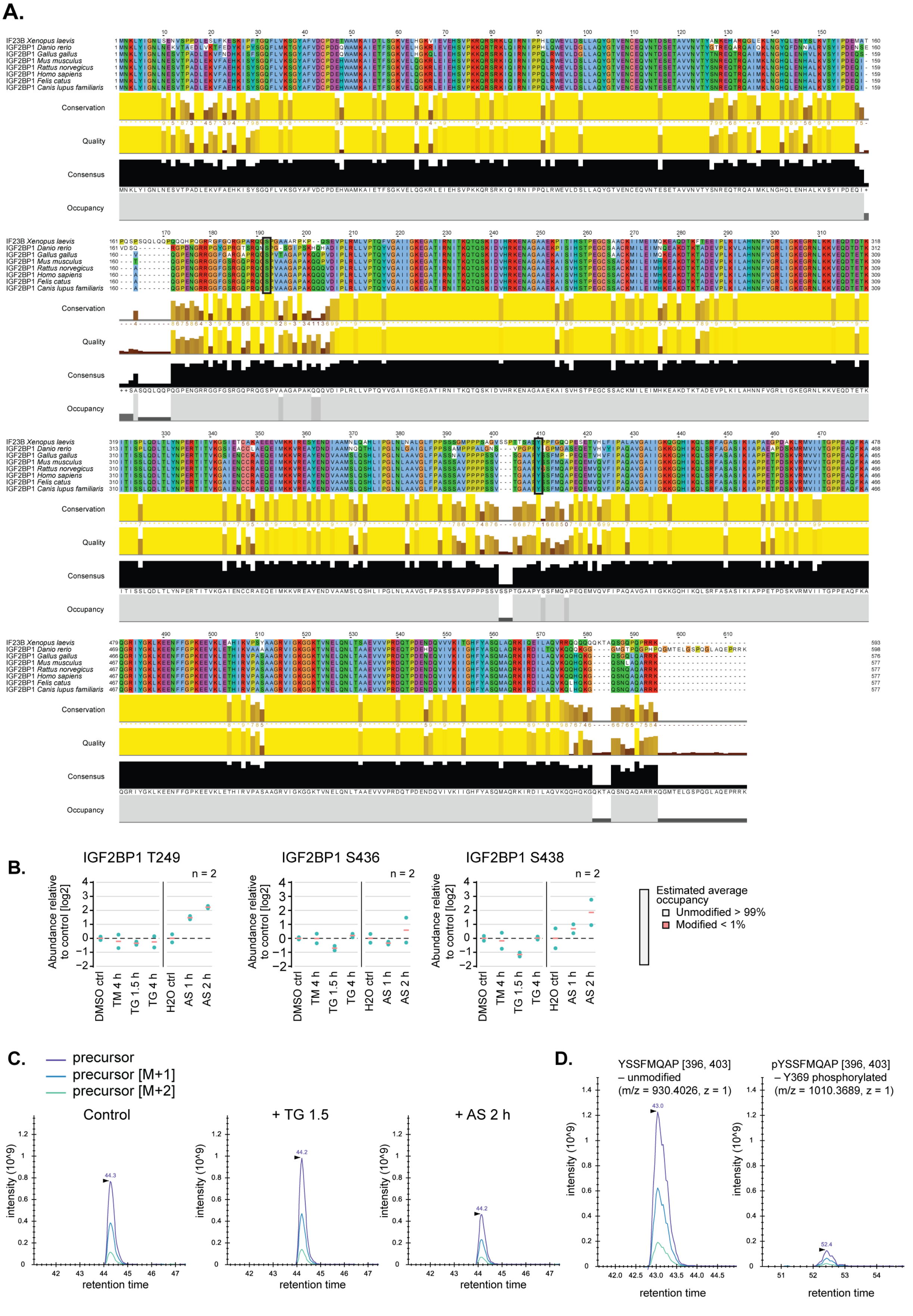

Supplementary Figure 1. IGF2BP1 phosphorylation sites S181 and Y396 are highly conserved
(A) Comparison of the IGF2BP1 primary protein sequence from*; Xenopus laevis; Danio rerio; Gallus gallus; Mus musculus; Rattus norvegicus; Homo sapiens; Felis catus and Canis lupus familiaris.* (B)

Relative abundance of the indicated IGF2BP1 phosphorylation sites in cells exposed to various forms of proteotoxic stress compared to the control conditions. Tunicamycin (TM) and thapsigargin (TG) induces ER stress, whereas sodium arsenite (AS) leads to oxidative stress. The time-points on the bottom indicate length of exposure to the stress-inducing drug. (C) Extracted ion chromatogram of LC-MS/MS peaks of the YSSFMQAP peptide obtained from IGF2BP1 isolated from mammalian cells under control, thapsigargin, and arsenite conditions (D) Extracted ion chromatogram of LC-MS/MS peaks of unmodified (left) and phosphorylated (right) YSSFMQAP peptides obtained from *in vitro* purified IGF2BP1 after ProAlanase treatment. The in vitro phosphorylation was performed by Src kinase treatment, please see materials and methods

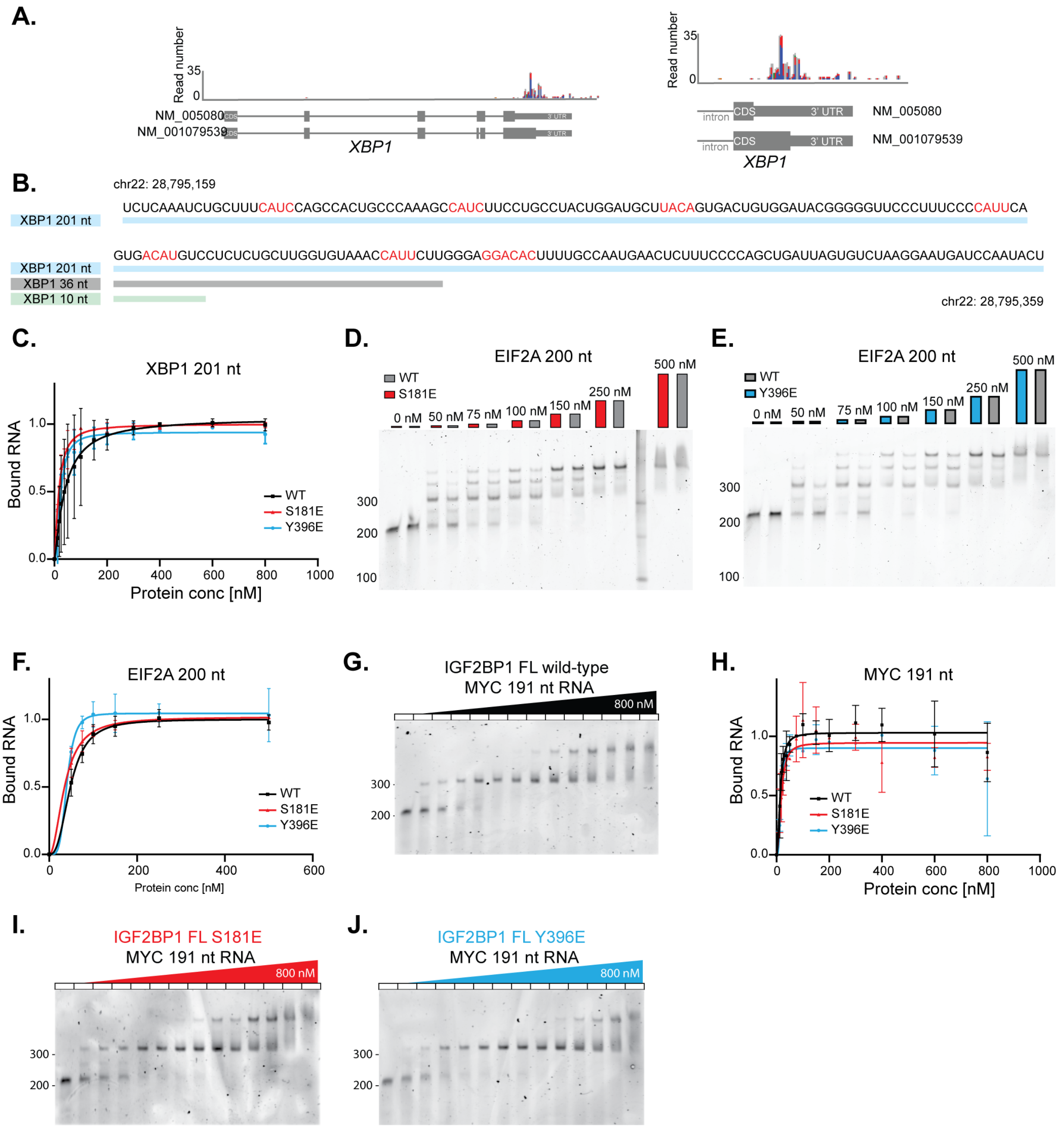

Supplementary Figure 2. Phosphomimetic mutations do not affect IGF2BP1 binding to model RNAs
(A) IGF2BP1 binding sites in XBP1 identified by analyzing PAR-CLIP data published by Hafner et al. 2010. (B) Sequence of the XBP1-derived RNAs: XBP1 201 nt (blue), XBP1 36 nt (grey) and XBP1 10 nt RNA (green) with genome annotation. Predicted RNA binding motifs are highlighted in red. (C) Quantification of EMSA assays with IGF2BP1 full-length wild-type (black), S181E (red) and Y396E (blue) and XBP1 201 nt RNA from Fig. 2 (A-C) in duplicates. RNA binding is represented by the depletion of free RNA. Mean intensity from 0 nM protein sample was used to define 0 while the mean intensity of gel background represents 1. Dose response equation was used for curve fitting and calculation of K_D. Error bars represent the standard deviation. (D) (E) EMSA assays assessing IGF2BP1 full-length wild-type (black), S181E (red) and Y396E (blue) interaction with EIF2A 200 nt RNA. (F) Quantification of EMSA assays of IGF2BP1 full-length wild-type (black), S181E (red) and Y396E (blue) with EIF2A 200 nt RNA from Fig. Supp. 2 (D, E) in duplicates. Dose response equation was used for curve fitting and calculation of $K_{1/2}$. Error bars represent the standard deviation. (G) EMSA assays assessing IGF2BP1 full-length wild-type (black) interaction with MYC 191. (H) Quantification of EMSA assays of IGF2BP1 full-length wild-type (black), S181E (red) and Y396E (blue) with MYC 191 nt RNA from Fig. Supp. 2 (G, I, J) in

duplicates as described in Supp. Fig. 2C. EMSA assays assessing (I) S181E (red) and (J) Y396E (blue) interaction with MYC 191 nt RNA.

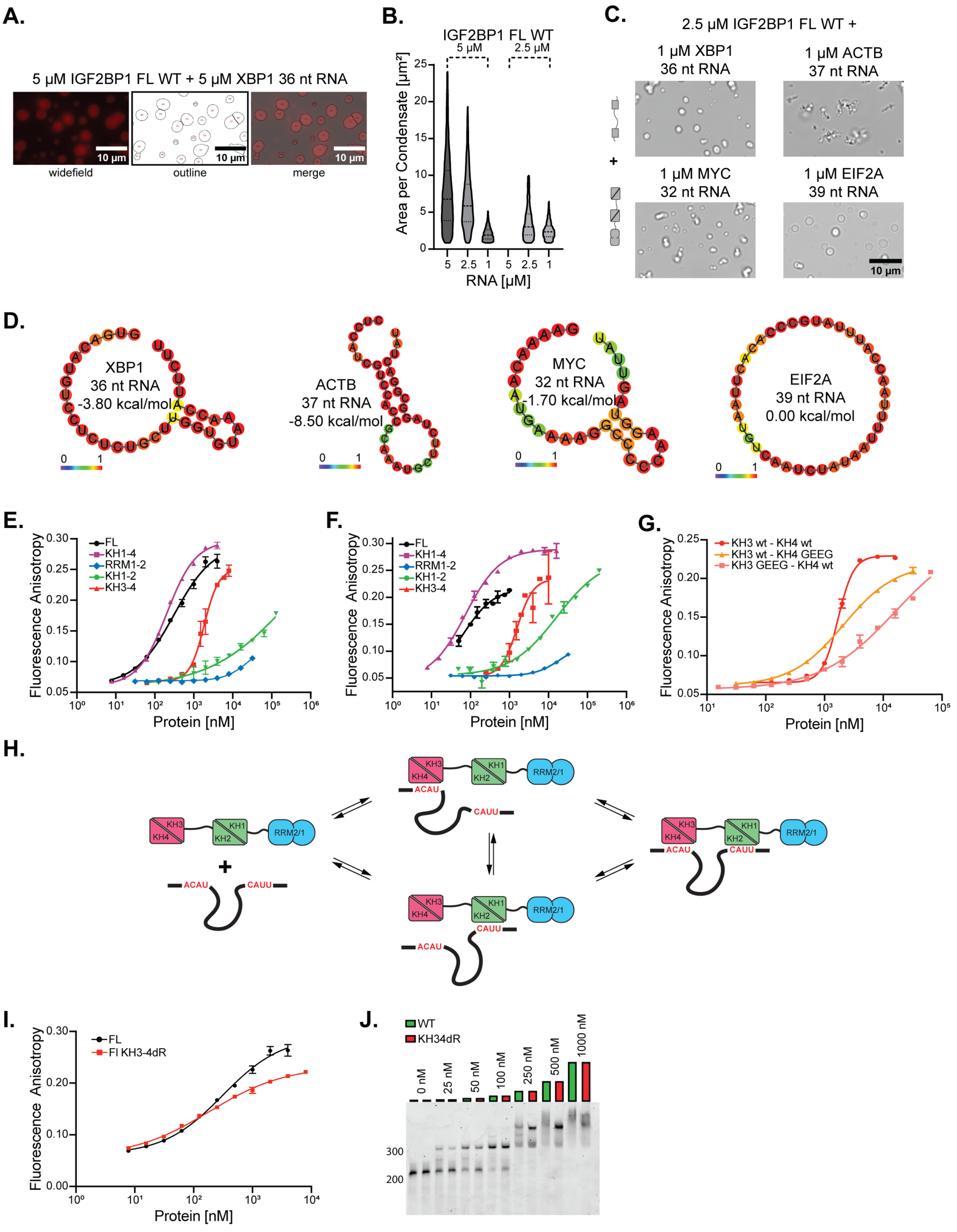

Supplementary Figure 3. KH3-4 domains drive RNA binding and RNP granule formation

(A) Representative images, outlines and overlay of the quantification of granules containing 5 µM IGF2BP1 with 5 % mCherry-IGF2BP1 and 5 µM XBP1 36 nt RNA after 90 min of incubation. (B) Violin plots of the area per condensate of IGF2BP1 full-length wild-type with XBP1 36 nt RNA at different protein and RNA concentrations. Dashed line represents median, the dotted lines 25 % and 75 % quartiles. (C) RNP granule formation assays of IGF2BP1 full-length wild-type with XBP1 36 nt, ACTB 37 nt, MYC 32 nt and EIF2A 39 nt RNA. Scale bar is 10 µm. Protein and RNA valency (left) is depicted as folded domains and the number of binding motifs. Secondary structure and minimum free energy thereof

predicted via the Vienna RNAfold Webserver [1] of the (D) XBP1 36 nt RNA, MYC 32 nt RNA, ACTB 37 nt RNA and EIF2A 39 nt RNA. (E) Fluorescence anisotropy assays (FA) to measure binding of full-length IGF2BP1 (black), IGF2BP1 KH1-4 (purple), IGF2BP1 RRM1-2 (blue), IGF2BP1 KH1-2 (green) and IGF2BP1 KH3-4 (red) to XBP1 36 nt RNA. X-axis in log-scale. (F) FA to measure binding of IGF2BP1 full-length and truncation mutants to ACTB RNA, the color-code is same as Supp. Figure 3D. (G) FA of IGF2BP1 KH3-KH4 (red), IGF2BP1 KH3-KH4GEEG (orange) and KH3GEEG-KH4 (pink) with XBP1 36 nt. X-axis in log-scale. (H) The model shows the avidity effect of IGF2BP1 interacting with XBP1-derived RNA. (I) FA of full-length IGF2BP1 (black) and full-length IGF2BP1 KH3GEEG-KH4GEEG (KH3-4dR, red) with XBP1 36 nt RNA. X-axis represented in log-scale. (J) EMSA assay of full-length IGF2BP1 (black) and full-length IGF2BP1 KH3GEEG-KH4GEEG (KH3-4dR, red) with XBP1 201 nt RNA. (I) RNP granule formation assays of 5 μM IGF2BP1 KH3-4 mutant KH3-GEEG and IGF2BP1 KH3-4 mutant KH4-GEEG with 5 μM XBP1 36 nt RNA.

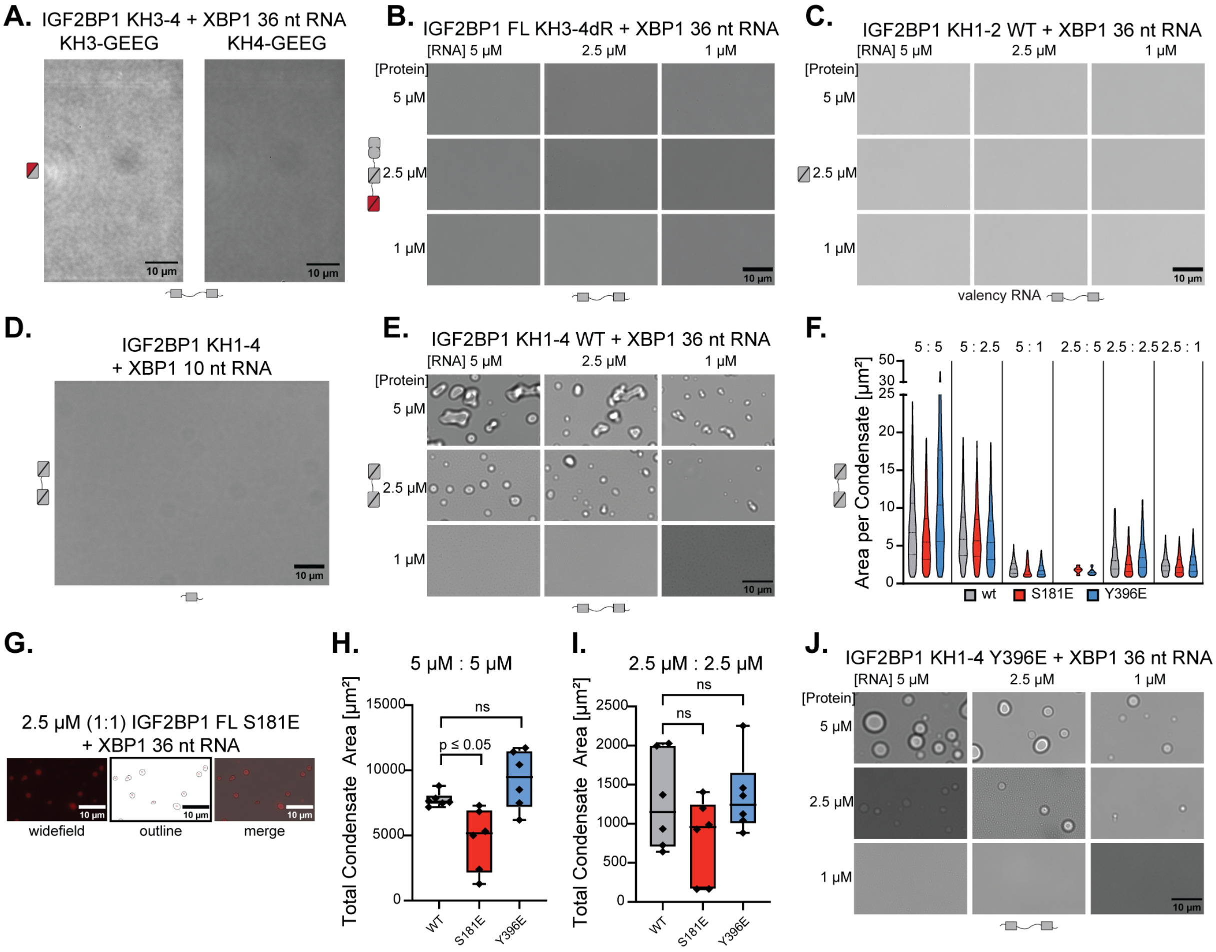

Supp. Figure 4. Mutantions affect granule formation of IGF2BP1
(A) RNP granule formation assays of 5 µM IGF2BP1 KH3-4 mutant KH3-GEEG and IGF2BP1 KH3-4 mutant KH4-GEEG with 5 µM XBP1 36 nt RNA. Protein (left) and RNA valency (bottom) is depicted as folded domains and the number of binding motifs. GEEG mutations indicated in red. (B) RNP granule formation assays of: IGF2BP1 full-length KH3-4dR (double mutant) with XBP1 36 nt RNA (C) 6XHis-IGF2BP1 KH1-2 and XBP1 36 nt RNA (D) 25 µM IGF2BP1 KH1-4 with 25 µM XBP1 10 nt RNA (30 min incubation) (E) IGF2BP1 KH1-4 wild-type and XBP1 36 nt RNA (250 mM NaCl). (F) Violin plots of the area per condensate for IGF2BP1 full-length wild-type, S181E and Y396E with XBP1 36 nt RNA at different protein and RNA concentrations. The dashed line represents the median value, the dotted lines the upper and lower quartiles. (G) Representative fluorescence microscopy images of condensates formed by full-length IGF2BP1 S181E, 5 % mCherry-IGF2BP1 S181E and XBP1 36 nt RNA (2.5 µM, 1:1). Box plots of total condensate area per field of view in (H) 5 µM protein + 5 µM RNA and (I) 2.5 µM protein + 2.5 µM RNA (see Supplementary Table 3). Two-tailed Welch's t-test was used for statistical analyses. (J) RNP granule formation assays of IGF2BP1 KH1-4 Y396E and XBP1 36 nt RNA (250 mM NaCl).

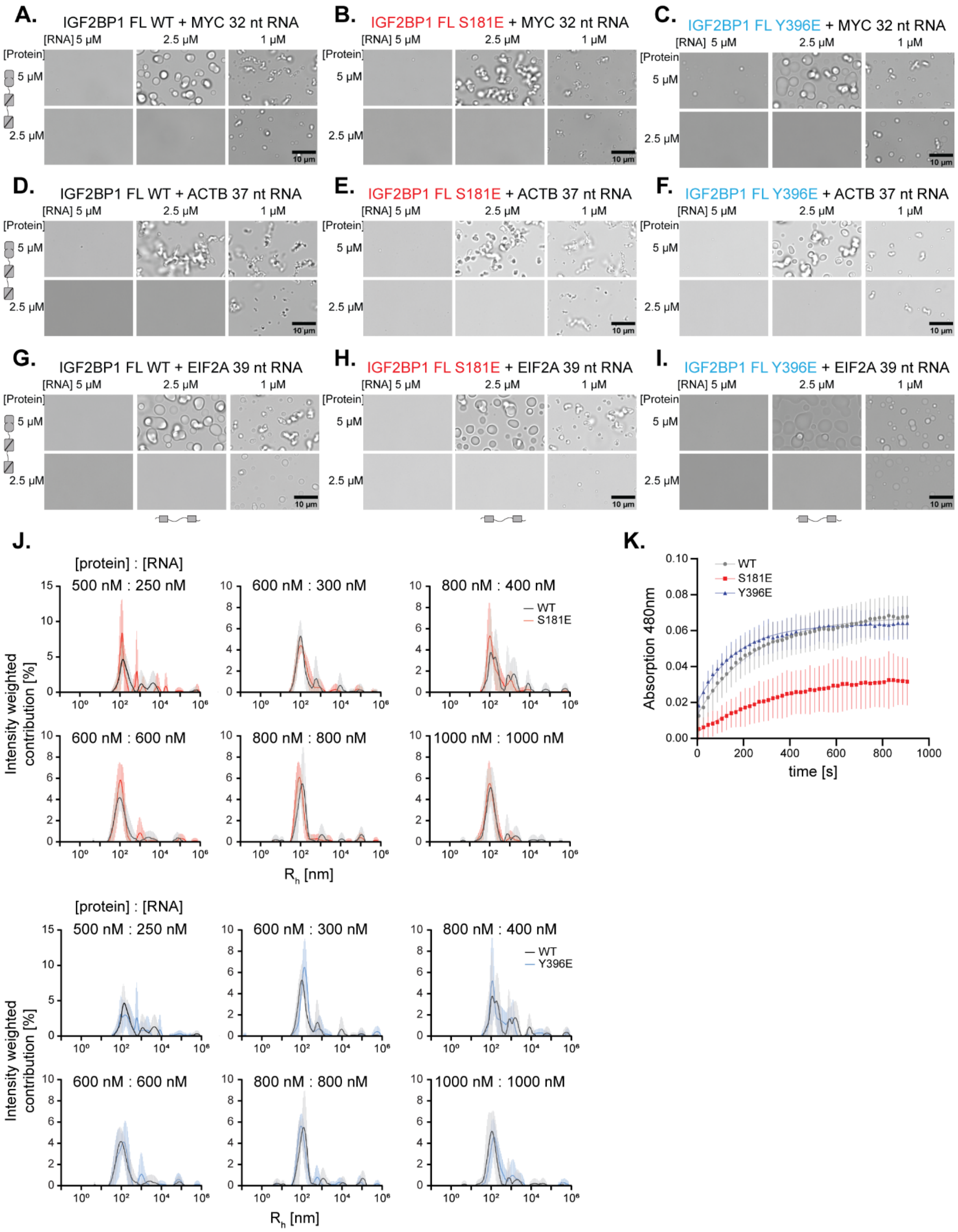

Supplementary Figure 5. IGF2BP1 phosphomimetic mutants display similar effects on RNP condensate formation in the presence of different model RNAs

(A) Brightfield images of IGF2BP1 wild-type, (B) S181E and (C) Y396E with MYC 32 nt RNA. Scale bar is 10 µm. (D) Brightfield images of IGF2BP1 wild-type, (E) S181E and (F) Y396E with ACTB 37 nt RNA. Scale bar is 10 µm. (G) Brightfield images of IGF2BP1 wild-type, (H) S181E and (I) Y396E with EIF2A 39 nt RNA. Scale bar is 10 µm. The valency (left, per row) is depicted as folded domains. (J) Distributions of hydrodynamic radii as their intensity weighted contribution in [%] of IGF2BP1 wild-type (black), S181E (red) and Y396E (blue) with XBP1 36 nt RNA at depicted protein and RNA concentrations. Data recorded in three independent experiments as technical triplicates. n is between 5 and 9, see Methods for details.

(K) Turbidity assay monitoring condensate formation kinetics at 5 µM IGF2BP1 full-length wild-type (grey), S181E (red) and Y396E (blue) and 5 µM XBP1 36 nt RNA. Error bars represent the standard deviation (WT n = 10, S181E n=11, Y396E n=10) . The data is fitted to a one-phase association equation.

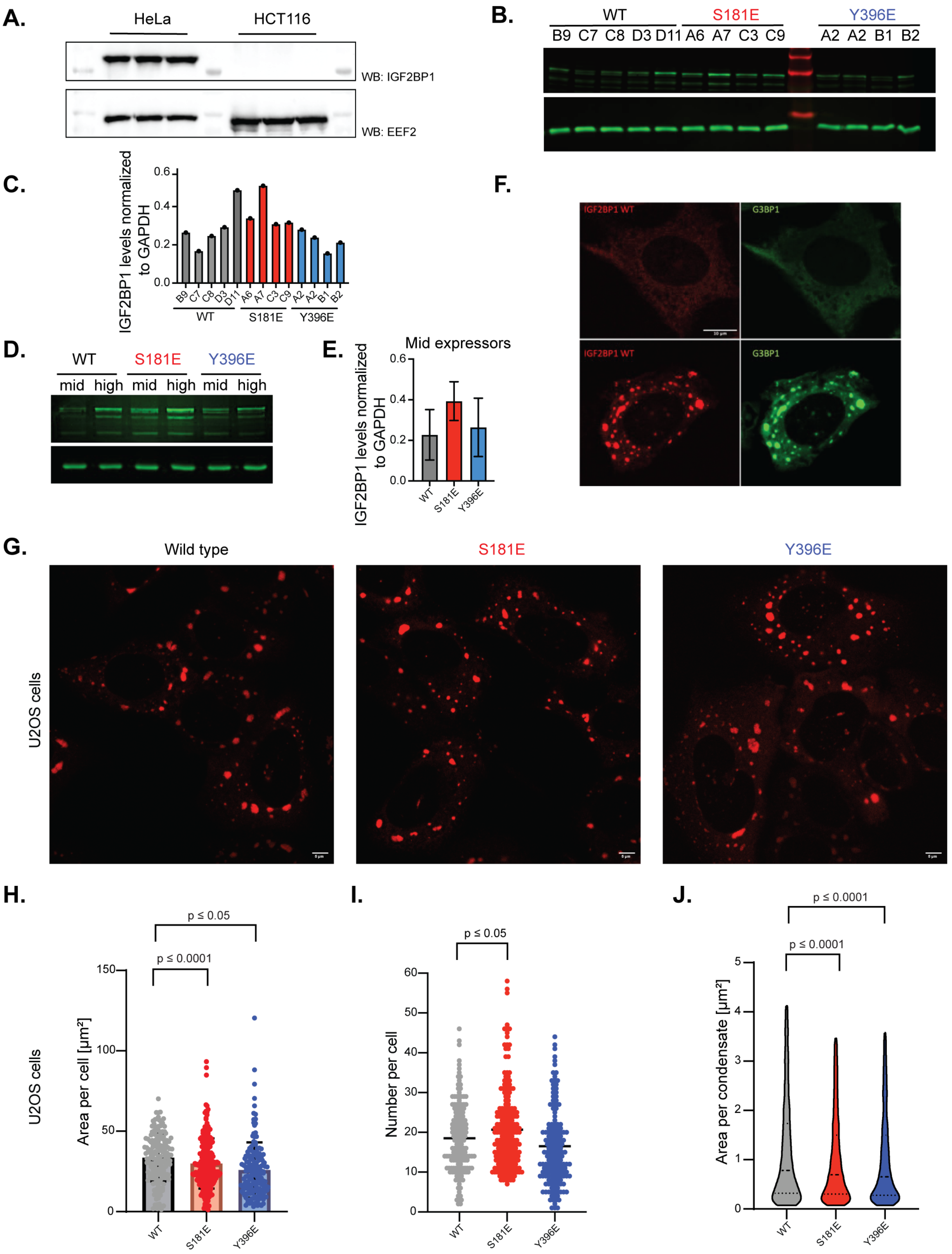

Supplementary Figure 6. IGF2BP1 phosphomimetic mutants affect granule sizes and numbers in cells (A) Western Blot analyses of IGF2BP1 expression in HeLa and HCT116 cells (Anti-IGF2BP1 antibody, MBL, RN001M; Anti-EEF2 antibody, Proteintech, 20107-1-AP). (B) Western Blot analyses of expression level of mCherry-IGF2BP1 wild-type, S181E and Y396E mutants expressed in single clone isolated from HCT116 cells by FACS. The IDs of clones are indicated on the WB. IGF2BP1 is stained by a fluorescently labeled anti-mCherry antibody. (C) Quantification of Western Blots from HCT116 cells. The IGF2BP1 band intensities were normalized to bands for the GAPDH control by the LI-COR system for

quantification. (D) Western Blot analyses of expression level of mCherry-IGF2BP1 wild-type, S181E and Y396E mutants expressed in U2OS cells. Mid and high indicate the expression levels identified by FACS. IGF2BP1 is stained by a fluorescently labeled anti-mCherry antibody. (E) Quantification of Western Blots from U2OS cells. The IGF2BP1 bands were normalized to GAPDH control. The LI-COR system was used for quantification. (F) Fluorescence microscopy images of fixed U2OS cells expressing mCherry-IGF2BP1 full-length wild-type and GFP-G3BP1 before (top row) and after (bottom row) treatment with 500 μM arsenite for 30 min. (G) Representative images of fluorescence microscopy from fixed U2OS cells expressing m-Cherry-IGF2BP1 full-length wild-type, m-Cherry-IGF2BP1 full-length S181E or m-Cherry-IGF2BP1 full-length Y396E stressed with 500 μM arsenite for 60 min. Scale bar is 5 μm. Quantification of condensates in U2OS cells represented as scatter plots: (H) total area of condensates per single cell (n = 162 for wild-type, n=230 for S181E, n=162 for Y396E; bar represents the mean value; two-tailed t-test was used to compare wild-type with S181E and Y396E) (I) number of condensates per single cell (bar represents the mean value; two-tailed t-test was used to compare wild-type with S181E and Y396E) and (J) area per condensate (the dashed line represents the median value, the dotted lines represent the 25 % and 75 % quartiles; two-tailed Mann-Whitney test was used to compare wild-type with S181E and Y396E).

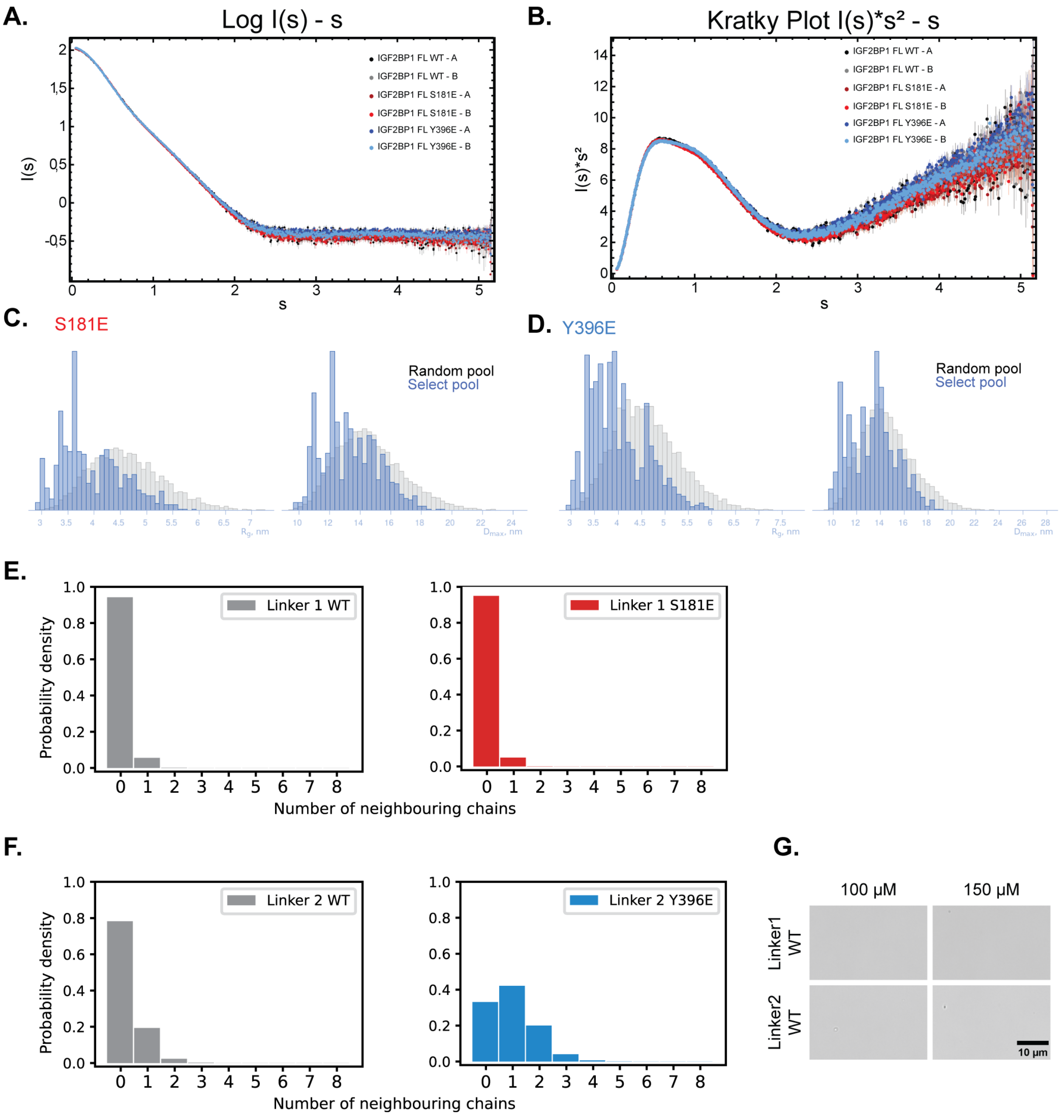

Supplementary Figure 7. IGF2BP1 forms a compact conformation in solution
(A) Intensity plot I(s) - s of SEC-SAXS data from IGF2BP1 full-length wild-type (black), IGF2BP1 full-length S181E (red), IGF2BP1 full-length Y396E (blue) in duplicates (A, B). Y-axis is represented in log_{10} scale. (B) Kratky plot I(s)*s² – s of SEC-SAXS data from IGF2BP1 full-length wild-type (black), IGF2BP1 full-length S181E (red), IGF2BP1 full-length Y396E (blue) in duplicates (A, B). (C) Comparison of R_g and D_{max} distribution of random conformations of IGF2BP1 S181E and selected pool that best fit the experimental SAXS data based on EOM analyses. (D) Comparison of R_g and D_{max} distribution of random conformations of IGF2BP1 Y396E and selected pool that best fit to the experimental SAXS data based on EOM analyses. (E) Probability distribution of pairwise contacts in MD simulations of Linker1 WT, Linker 1 S181E as well as (F) Linker2 WT and Linker2 Y396E. (G) Brightfield images of linker 1 (100 µM) and linker 2 (150 µM) and 15 % PEG8000 after 60 min incubation. Scale bar = 10 µm.

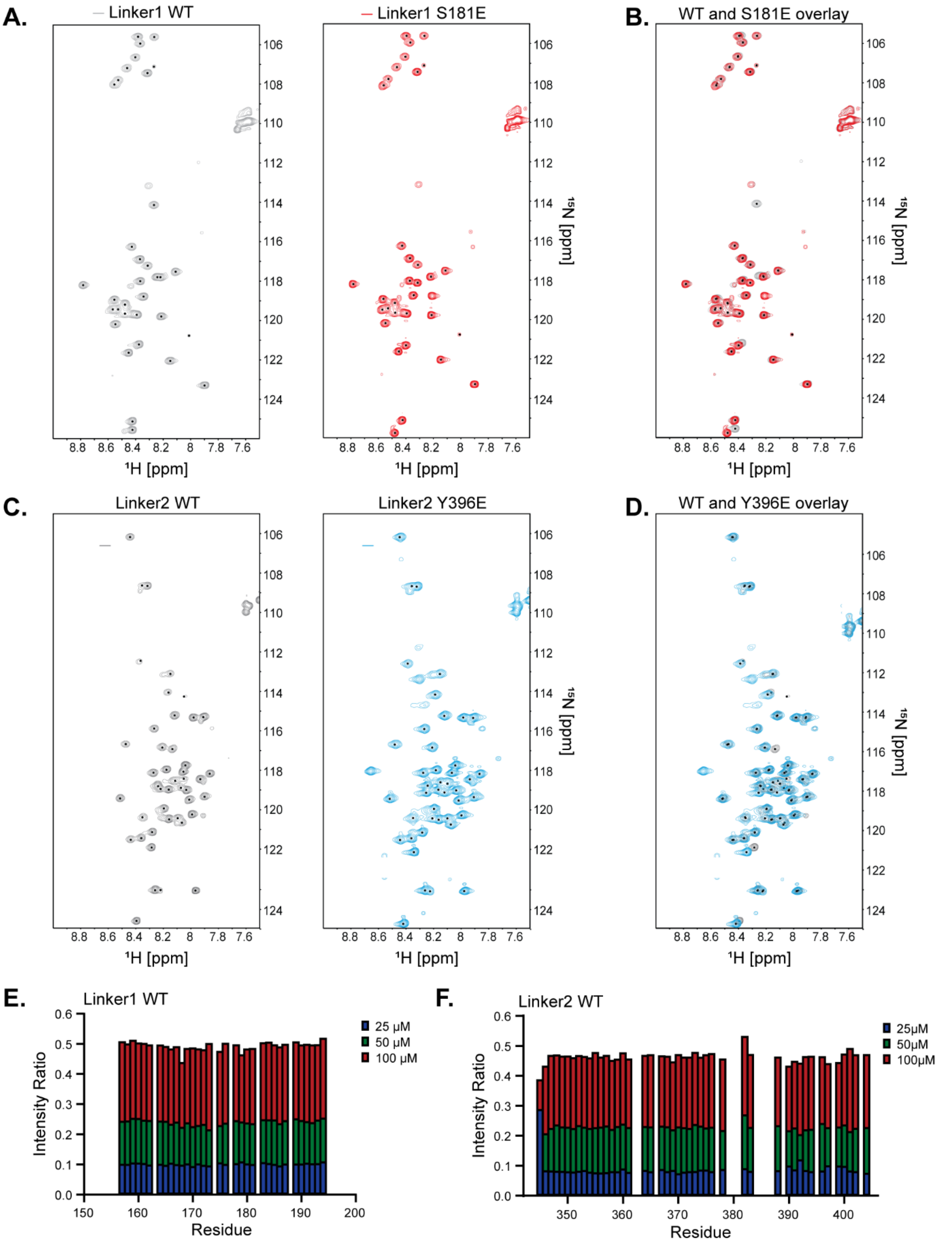

Supp. Figure 8. IGF2BP1 linkers do not strongly self-associate in solution
(A) HSQC spectra of ^{15}N-labeled linker 1 wild-type (grey) and ^{15}N-labeled linker 1 S181E (red) at 200 μM. Black dots represent assigned peaks. (B) Overlay of the HSQC spectra of linker 1 wild-type and linker 1 S181E (red). (C) HSQC spectra of ^{15}N-labeled linker 2 wild-type (grey) and ^{15}N-labeled Linker 2 Y396E (blue) at 200 μM. (D) Overlay of the HSQC spectra of linker 2 wild-type and linker 2 Y396E (blue). Black dots represent assigned peaks. (E) Plot of the intensity ratios of 100 μM, 50 μM and 25 μM to 200

μM ^{15}N-labeled linker 1 wild-type in HSQC. (F). Plot of the intensity ratios of 100 μM, 50 μM and 25 μM to 200 μM ^{15}N-labeled linker 2 wild-type in HSQC.

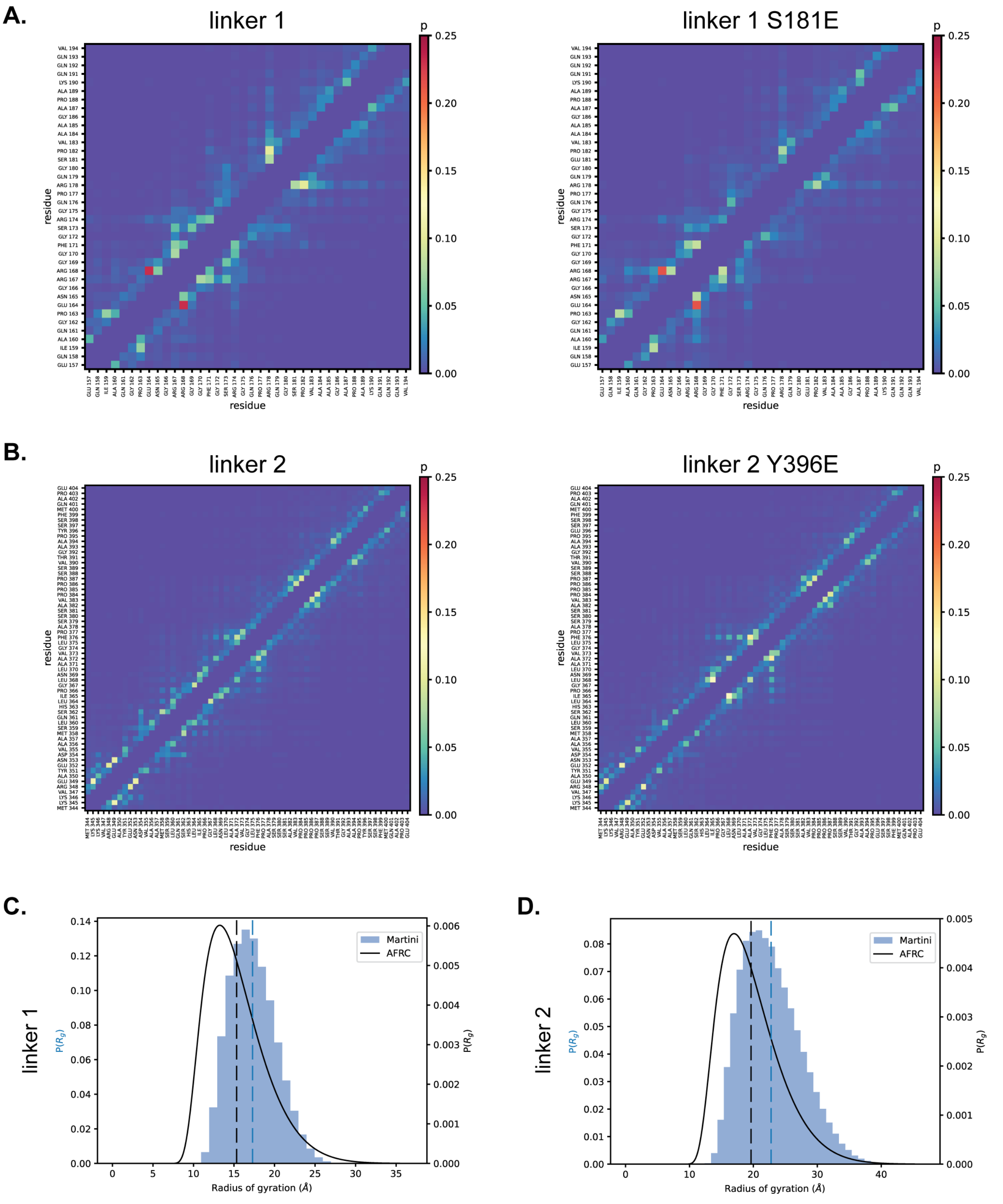

Supp. Figure 9. IGF2BP1 linkers do not display strong intermolecular interactions

(A) Contact map for cis-interactions of linker 1 and its phosphomimetic mutant S181E during MD simulations. (B) Contact map for cis-interactions of linker 2 and its phosphomimetic mutant Y396E during MD simulations. Value p measures the frequency of contact formation over the whole MD simulation. (C) Rg probability distribution of linker 1 and (D) linker 2 (filled blue steps) overlayed to the probability distribution computed for an ideal polypeptide with the same amino acidic sequence (black line). Dashed lines indicate the mean of the distribution.

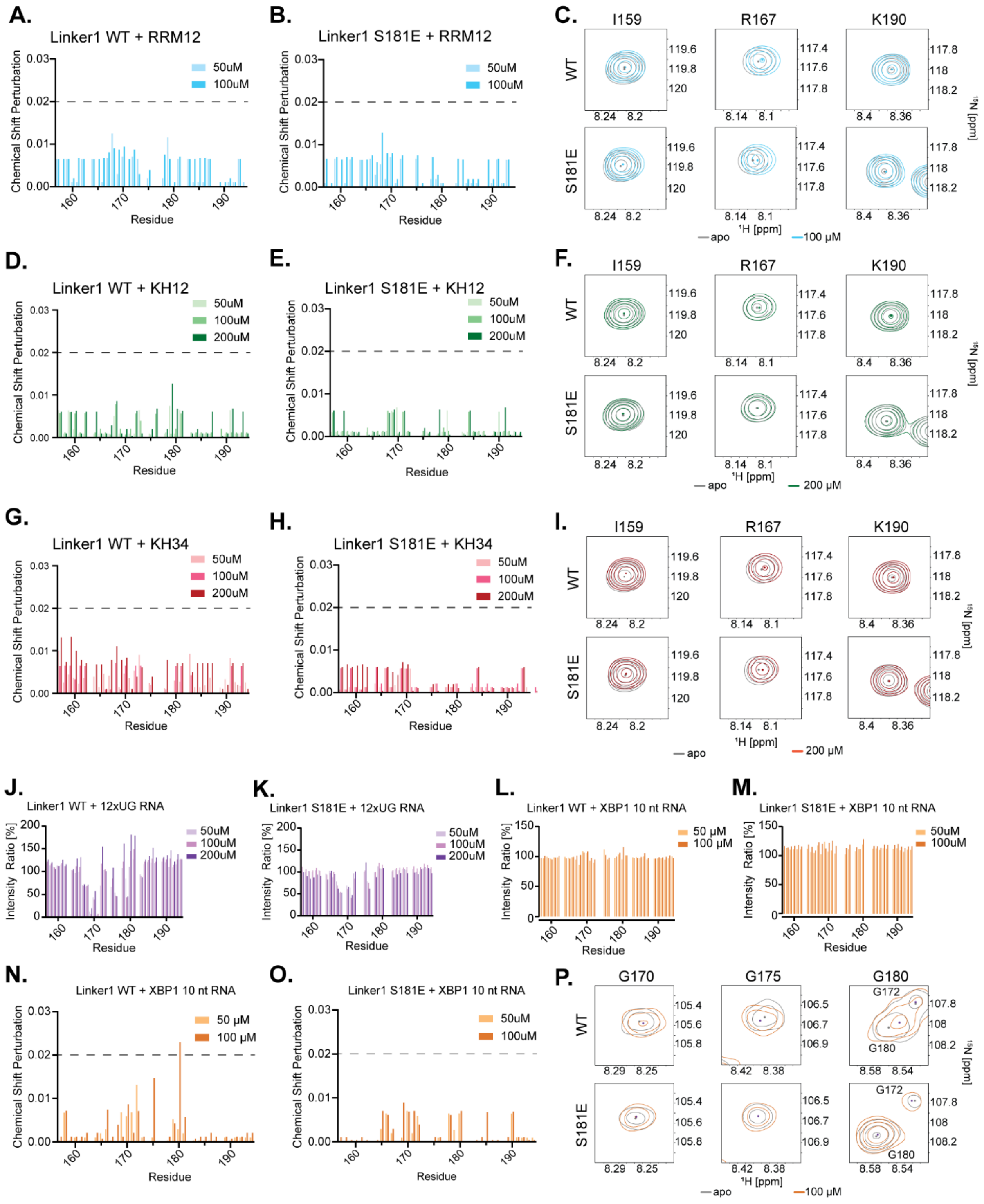

Supplementary Figure 10. Phosphomimetic mutation S181E in the linker1 affects low-affinity RNA interactions

(A) CSP analyses of ^{15}N-labeled linker 1 wild-type in the absence and presence of different concentrations of RRM1-2. (B) CSP analyses of ^{15}N-labeled linker 1 S181E with different concentrations of RRM1-2 (C) Representative HSQC signals in the ^{15}N-labeled linker 1 wild-type and S181E in the absence and presence of 100 µM RRM1-2 (D) CSP analyses of ^{15}N-labeled linker 1 wild-type with different concentrations of KH1-2. (E) CSP analyses of ^{15}N-labeled linker 1 S181E with different concentrations of KH1-2. (F) Representative peaks of ^{15}N-labeled linker 1 wild-type and S181E in the absence and presence of 200 µM KH1-2. (G) CSP analyses of ^{15}N-labeled linker 1 wild-type with different concentrations of KH3-4. (H) CSP analyses of ^{15}N-labeled linker 1 S181E with different concentrations of KH3-4. (I) Representative HSQC signals from ^{15}N-labeled linker 1 wild-type and

S181E in the absence and presence 200 μM KH3-4. (J) Intensity plots of signals in ^{15}N-labeled linker 1 wild-type in the presence of different concentrations of 12xUG RNA in HSQC normalized to signals in the apo spectrum. (K) Intensity plots of ^{15}N-labeled linker 1 S181E with different concentrations of 12xUG RNA in HSQC normalized to signals in the apo spectrum. (L) Intensity plots of ^{15}N-labeled linker 1 wild-type with different XBP1 10 nt RNA concentrations in HSQC normalized to signals in the apo spectrum. (M) Intensity plots of ^{15}N-labeled Linker 1 S181E with different concentrations of XBP1 10 nt RNA in HSQC normalized to apo spectrum. (N) CSP analyses of ^{15}N-labeled Linker 1 wild-type with different concentrations of XBP1 10 nt RNA. (O) CSPs of ^{15}N-labeled linker 1 S181E with different concentrations of XBP1 10 nt RNA. (P) Representative peaks of ^{15}N-labeled linker 1 wild-type and S181E in the absence and presence of 100 μM of XBP1 10 nt RNA.

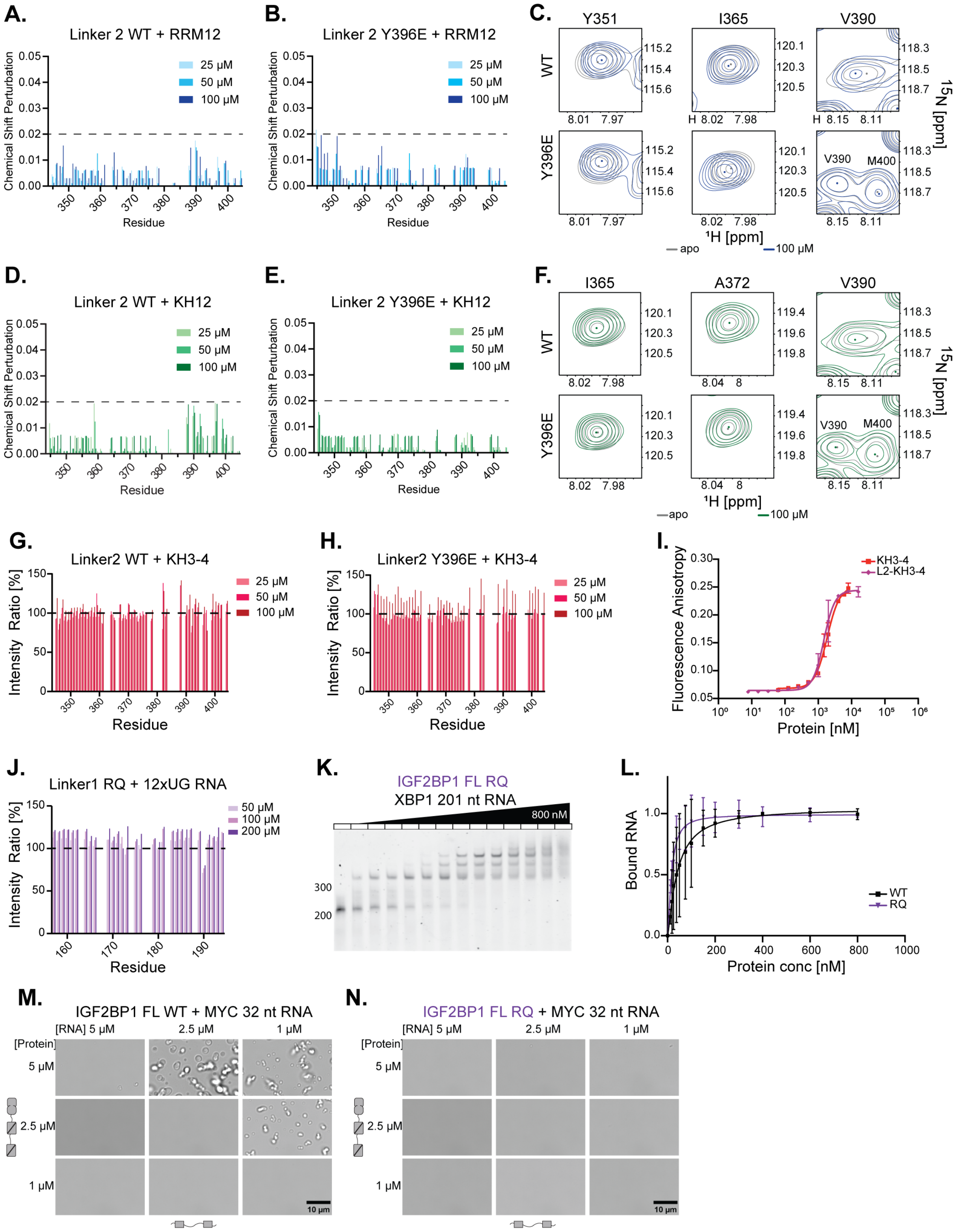

Supp. Figure 11. RGG motif in Linker 1 mediates low-affinity RNA interactions and regulates condensate formation

(A) CSP analyses of ^{15}N-labeled linker 2 wild-type in the absence and presence of various concentrations of RRM1-2 determined. (B) CSP analyses of ^{15}N-labeled linker 2 Y396E with different concentrations of RRM1-2. (C) Representative HSQC signals in ^{15}N-labeled linker 2 wild-type and Y396E in the absence and presence of 100 μM RRM1-2. (D) CSP analyses of ^{15}N-labeled linker 2 wild-type with different concentrations of KH1-2 (E) CSP analyses of ^{15}N-labeled linker 2 Y396E with different concentrations of KH1-2 (F) Representative HSQC signals in ^{15}N-labeled linker 2 wild-type and Y396E in the absence and presence of 100 μM KH1-2 (G) Intensity plots of ^{15}N-labeled wild-type linker 2 in the presence of different concentrations of KH3-4 normalized to linker 2 signals in the apo spectrum. (H)

Intensity plots of ^{15}N-labeled linker 2 Y396E in the presence of different concentrations of KH3-4 normalized to linker 2 Y396E signals in the apo spectrum. (I) Fluorescence anisotropy assays to assess binding of IGF2BP1 KH3-4 wild-type (red) and IGF2BP1 linker 2 KH3-4 wild-type (purple) to 5'-fluorescein labeled XBP1 36 nt. X-axis represented in log-scale. (J) Intensity plots of ^{15}N-labeled linker 1 RQ in the presence of different concentrations of 12x UG RNA in HSQC normalized to linker 1 RQ signals in the apo spectrum. (K) Electrophoretic Mobility Shift Assay (EMSA) to assess the binding of IGF2BP1 RQ with XBP1 201 nt RNA at concentrations ranging from 0 to 800 nM. (L) Quantification of EMSA assays of IGF2BP1 full-length wild-type (black) (from Fig. 2A) and IGF2BP1 RQ (purple) with XBP1 201 nt RNA (from Supplementary Figure 9K) in duplicates. Dose response equation was used for curve fitting and calculation of $K_{1/2}$. Error bars represent the standard deviation. (M) Bright field images of condensates formed by IGF2BP1 wild-type and (N) IGF2BP1 RQ with MYC 32 nt RNA. Scale bar is 10 µm. The valency of the protein (left) is depicted by the number of folded domains. The valency of the RNA (bottom) is depicted as the number and position of binding motifs.

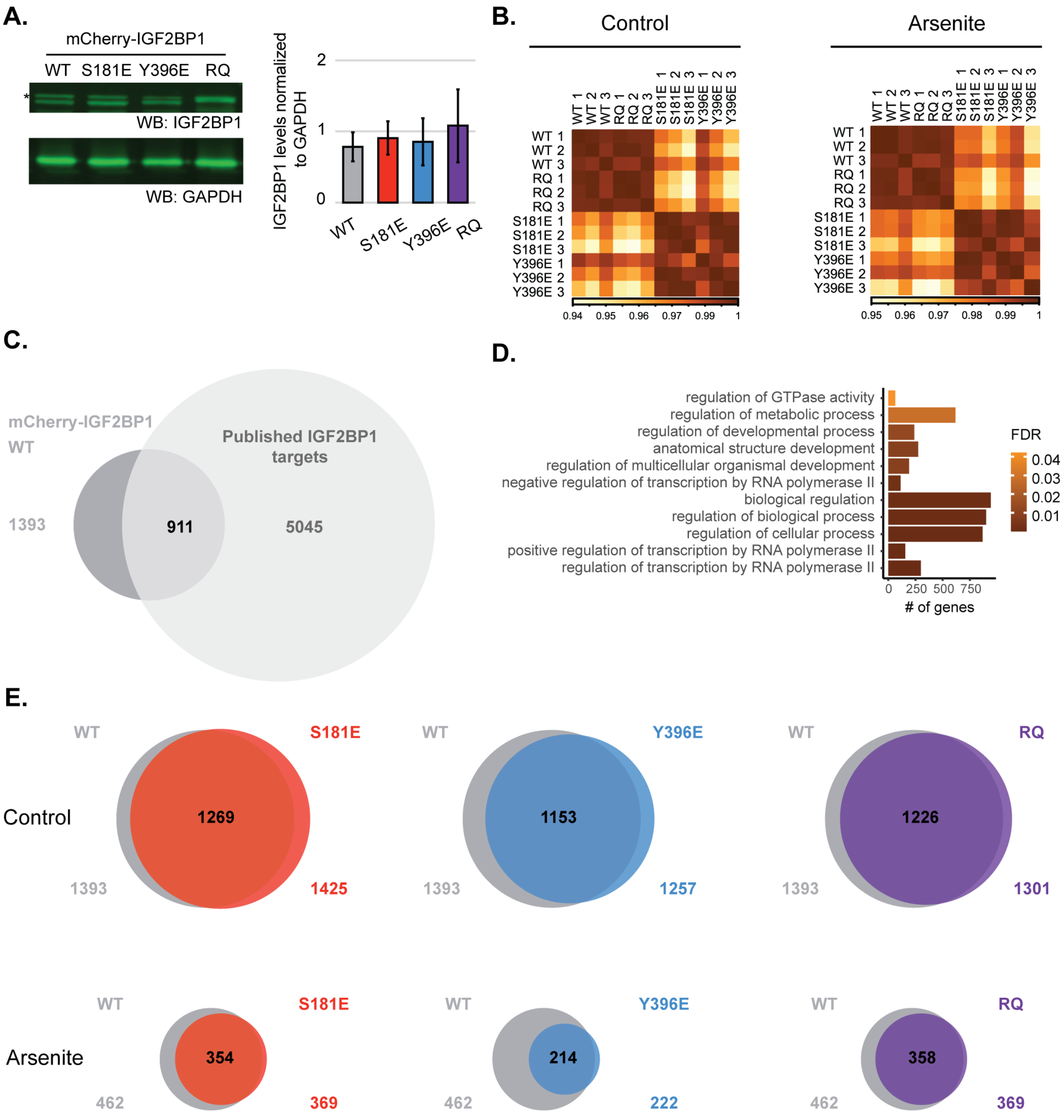

Supplementary Figure 12. Total transcriptome (RNA-Seq) and RNA immunoprecipitation sequencing (RIP-seq) of IGF2BP1 S181E, Y396E, and RQ mutants.
(A) Western Blot analyses of expression level of mCherry-IGF2BP1 wild-type and S181E, Y396E, and RQ mutants in a FACS-sorted narrow gate population of HCT116 cells used for transcriptomics and RIP-Seq experiments. n=3 technical replicates, data are presented as mean values +/- SD. (B) Pearson correlation matrices of total transcriptome of HCT116 cells expressing mCherry-IGF2BP1 wild-type or S181E, Y396E, and RQ mutants. Arsenite stress was induced with 2-hour treatment with 500 μM of sodium arsenite. (C) Venn diagram showing the intersection between the mRNA transcript targets identified in this study in control conditions for mCherry-IGF2BP1 (2-fold enrichment of RIP-Seq CPM compared to RNA-Seq CPM and 4-fold enrichment of RIP-Seq CPM compared to the background RIP-Seq (parental HCT116 cells) and the reported IGF2BP1 targets (PAR-CLIP PARalyzer-identified targets from HEK293 cells [2] with more than 5 binding sites [3]) (D) GO-term biological process enrichment analysis of mCherry-IGF2BP1 mRNA transcript targets identified in this study in control conditions. For visualization purposes the list of GO term was simplified using REVIGO [4] .(E) Venn diagrams showing

the intersection between the mRNA transcript targets identified for mCherry-IGF2BP1 wild-type and S181E, Y396E, and RQ mutants. Source data are available in Supplementary File 2.

	Sequence
XBP1 201 nt	UCUCAAAUCUGCUUU**CAUC**CAGCCACUGCCCAAAGC**CAUC**U<br>UCCUGCCUACUGGAUGCU**UACA**GUGACUGUGGAUACGGGG<br>GUUCCCUUUCCC**CAUU**CAGUG**ACAU**GUCCUCUCUGCUUGG<br>UGUAAAC**CAUU**CUUGGGA**GGAC**ACUUUUGCCAAUGAACUCU<br>UUCCCAGCUGAUUAGUGUCUAAGGAAUGAUCCAAUACU
EIF2A 200 nt	AGAAAAUUCAGAAAGA**AACA**GCCCUUCUCCAGGAGCUGGAA<br>GAUUUGGAAUUGGGUAUUUAAAGAUUCACGGAAAGCAAGUU<br>GAUGACCAGAAAUCAGUGCAAA**CACAU**CUUCUGUUAAACC**C**<br>**AUU**GGUA**UACACA**GAAUAUUCCUGUGCC**CACA**CUUAAUGUC<br>AAUCUAUAAUUUUAAC**CAUU**UAUCCAAGAUUCUACU
MYC 191 nt [8]	UGCGUGACCAGAUCCCGGAGUUGGAA**AACA**AUGAAAAGGCC<br>CCCAAGG**UAGU**UAUCCUUAAAAAAGC**CACA**G**CAUACAUC**CU<br>GUCCGUCCAAGCAGAGGAGCAAAAGCU**CAUU**UCUGAAGA**GG**<br>**AC**UUGUUGCGGAAACGACGAG**AACAGU**UGA**AACACA**AACUU<br>G**AACA**GCUACGGAACUCUUGUGCGUAA
XBP1 36 nt	GUG**ACAU**GUCCUCUCUGCUUGGUGUAAAC**CAUU**CUU
XBP1 10 nt	GUG**ACAU**GUC
MYC 32 nt [9]	GAA**AACA**AUGAAAAGGCCCCCAAGG**UAGU**UAU
ACTB 28 nt [10]	ACC**GGAC**UGUUACCAACACC**CACA**CCCC
ACTB 37 nt [11]	CUC**CAUC**GUCCACCGCAAAUGCUUCUAGGC**GGAC**UAU
EIF2A 39 nt	GCC**CACA**CUUAAUGUCAAUCUAUAAUUUUAAC**CAUU**UAU
12xUG	UGUGUGUGUGUGUGUGUGUGUGUG

Supplementary Table 1: Sequences of model RNAs

RNAs were used in the following experiments: Fig. 2A-M, Fig. 5H-M, Fig. Supp. 2C-J, Fig. Supp. 3A-G, I, J, Fig. Supp. 4A-J, Fig. Supp. 5A-K, Fig. Supp. 10J-P, Fig. Supp. 11I-N. Putative binding sites in **bold** [5-8].

EMSA			
XBP1 201 nt RNA	$K_{1/2}$ [nM]	95% CI	R^2
wild-type	41.01	20.87 to 174.8	0.7774
S181E	17.06	14.33 to 20.16	0.9727
Y396E	22.91	19.05 to 27.81	0.9519
RQ	17.00	12.08 to 22.95	0.9114
KH34dR	64.74	43,42 to 131,4	0,9739
EIF2A 200 nt RNA	$K_{1/2}$ [nM]	95% CI	R^2
wild-type	48.19	42.00 to 53.61	0.9686
S181E	35.42	23.25 to 43.23	0.9869
Y396E	40.17	14.90 to ???	0.9585
MYC 191 nt RNA	$K_{1/2}$ [nM]	95% CI	R^2
wild-type	14.36	8.902 to 20.02	0.831
S181E	17.78	10.76 to 27.61	0.7757
Y396E	16.70	12.31 to 21.90	0.8105

Fluorescence Anisotropy					
XBP1 36 nt RNA	$K_{1/2}$[nM]	95% CI	Hill-Coeff.	95% CI	R^2
wild-type	311.7	258.2 to 390.5	0.8640	0.7525 to 0.9900	0.9932
S181E	310.1	256.3 to 377.5	1.049	0.8728 to 1.258	0.9855
Y396E	423.3	344.8 to 530.7	0.9587	0.7963 to 1.150	0.9862
RQ	113.8	97.76 to 132.7	0.8618	0.7680 to 0.9690	0.9928
KH1-4 wild-type	204.0	188.4 to 221.6	1.174	1.073 to 1.285	0.9984
RRM1-2 wild type	25928	13310 to ???	1.410	0.8336 to 2.258	0.9340
KH1-2 wild-type*	-	-			-
KH3-4 wild-type	1850	1661 to 2151	2.338	1.741 to 3.112	0.9832
L2-KH3-4 wild-type	1470	1318 to 1640	2.620	2.042 to 3.392	0.9809
Full-length KH3 GEEG KH4 GEEG	185.8	157.4 to 223.2	0.6683	0.6089 to 0.7328	0.9957
KH3 wild-type KH4 GEEG	2.122	1.980 to 2.286	0.9702	0.9142 to 1.029	0.9988
KH3 GEEG KH4 wild-type	15.69	9.738 to 33.85	0.6979	0.5827 to 0.8249	0.9906
ACTB 28 nt RNA	$K_{1/2}$[nM]	95% CI	Hill-Coeff.	95% CI	R^2
Full-length wild-type	68.55	56.88 to 87.17	1.116	0.7966 to 1.471	0.9818
KH1-4 wild-type	75.18	65.69 to 86.12	0.9804	0.8803 to 1.096	0.9953
RRM1-2 wild type	19839	11390 to 287085	1.368	0.8564 to 2.067	0.9449
KH1-2 wild-type	16670	10009 to 44411	0.7285	0.5232 to 0.9849	0.9793
KH3-4 wild-type	1574	1175 to 2575	2.109	1.024 to 4.634	0.9285

Supplementary Table 2: Fit-values of binding experiments

$K_{1/2}$ values of different full-length IGF2BP1 constructs binding to different RNAs from EMSA and Fluorescence Anisotropy assays in Fig. 2A-D, Fig. Supp. 2C-J, Fig. Supp. 3E-G, I J, Fig. Supp. 11I, K, L; *not enough data points for a reasonable fit

	Median area per condensate [µm²]	25% Percentile	75% Percentile	Mean total area of condensates [µm²]
IGF2BP1 wild-type	7.022	3.955	11.28	7753
IGF2BP1 S181E	5.716	3.306	9.342	4691
IGF2BP1 Y396E	10.790	5.758	18.52	9298

Supplementary Table 3: Quantification of *in vitro* RNP granule formation
Area per condensate of IGF2BP1 full-length constructs with XBP1 36 nt RNA (5 µM protein plus 5 µM RNA) in Fig. 2J and total condensate area in the same conditions (Fig. Supp. 4H).

	$t_{1/2}$ [s]	95% CI	OD_{480}	95% CI	R^2
IGF2BP1 wild-type	167	138.4 to 205.7	0.06775	0.06531 to 0.07082	0.6448
IGF2BP1 S181E	251.6	171.8 to 431.4	0.03505	0.03130 to 0.04282	0.3264
IGF2BP1 Y396E	115.3	97.98 to 136.8	0.06358	0.06230 to 0.06501	0.6495

Supplementary Table 4: Turbidity curve over 15 min of different IGF2BP1 constructs with XBP1 36 nt RNA based on Fig. Supp. 5K.

	Dynamic pop. [%]	95% CI	$t_{1/2}$ [s]	95% CI	R^2
IGF2BP1 wild-type	68.81	67.35 to 70.46	21.58	19.44 to 24.09	0.5531
IGF2BP1 S181E	62.15	60.04 to 64.70	37.46	33.58 to 42.22	0.6744
IGF2BP1 Y396E	74.07	73.75 to 74.40	15.50	15.04 to 15.98	0.9079

Supplementary Table 5: The analyses of the FRAP curves of different IGF2BP1 constructs in G3BP1 induced SGs in Fig. 3D.

HCT116 cells	Median area per condensate [µm²]	25% Percentile	75% Percentile	Total number of condensates
wild-type	1.142	0.4985	2.286	5272
S181E	1.026	0.4404	2.148	6719
Y396E	1.093	0.4336	2.248	3927
RQ	1.276	0.5518	2.217	3826
HCT116 cells	Median total area per cell [µm²]	25% Percentile	75% Percentile	Mean number of condensates per cell
wild-type	10.26	6.907	13.55	5.71
S181E	10.26	6.496	14.82	7.205
Y396E	10.15	6.811	15.22	6.291
RQ	8.648	6.011	11.67	5.927
U2OS cells	Median area per condensate [µm²]	25% Percentile	75% Percentile	Total number of condensates
wild-type	0.976	0.369	2.408	2980
S181E	0.824	0.333	1.981	4463
Y396E	0.803	0.304	2.097	2685
U2OS cells	Median total area per cell [µm²]	25% Percentile	75% Percentile	Mean number of condensates per cell
wild-type	34.19	22.87	44.3	18.54
S181E	27.12	19.68	39.74	20.66
Y396E	22.79	13.53	33.97	16.52

Supplementary Table 6: Area per condensate of IGF2BP1 full-length constructs expressed in HCT116 and U2OS cells based on experiments in Fig.3 E-H, Fig. Supp. 6G-J.

	Dynamic pop. [%]	95% CI	$t_{1/2}$ [s]	95% CI	R^2
IGF2BP1 wild-type	77.86	75.54 to 80.56	241.7	221.1 to 266.0	0.801
IGF2BP1 S181E	61.32	59.37 to 63.61	240.0	217.8 to 266.6	0.7869
IGF2BP1 Y396E	69.36	67.90 to 70.96	151.3	138.1 to 166.5	0.7347

Supplementary Table 7: The analyses of the FRAP curves of different IGF2BP1 constructs in U2OS cells (Fig. 3I).

	Method	<Rg> (Å)	<Ree> (Å)
Linker 1 WT	CALVADOS	16.3 ± 0.1	39.1 ± 0.4
Linker 1 WT	Martini	17.3	-
Linker 1 WT	AFRC	15.31	35.81
Linker 1 S181E	CALVADOS	16.25± 0.09	39.0 ± 0.4
Linker 1 S181E	Martini	17.4	-
Linker 1 S181E	AFRC	15.32	35.8
Linker 2 WT	CALVADOS	22.3 ± 0.1	52.7 ± 0.7
Linker 2 WT	Martini	22.8	-
Linker 2 WT	AFRC	19.63	45.76
Linker 2 Y396E	CALVADOS	22.2 ± 0.1	52.7 ± 0.6
Linker 2 Y396E	Martini	22.5	-
Linker 2 Y396E	AFRC	19.62	45.75

Supplementary Table 8: The radius of gyration values of the linkers and their mutants based on the MD simulations in Fig. 4C-F.

	Sequence
Full-length wild-type	GPLGSPGIPGMNKLYIGNLNESVTPADLEKVFAEHKISYSGQFLVKSGYAF<br>VDCPDEHWAMKAIETFSGKVELQGKRLEIEHSVPKKQRSRKIQIRNIPPQL<br>RWEVLDSLLAQYGTVENCEQVNTESETAVVNVTYSNREQTRQAIMKLNG<br>HQLENHALKVSYIPDEQIAQGPENGRRGGFGSRGQPRQGSPVAAGAPAK<br>QQQVDIPLRLLVPTQYVGAIIGKEGATIRNITKQTQSKIDVHRKENAGAAEK<br>AISVHSTPEGCSSACKMILEIMHKEAKDTKTADEVPLKILAHNNFVGRLIGK<br>EGRNLKKVEQDTETKITISSLQDLTLYNPERTITVKGAIENCCRAEQEIMKK<br>VREAYENDVAAMSLQSHLIPGLNLAAVGLFPASSSAVPPPPSSVTGAAPYS<br>SFMQAPEQEMVQVFIPAQAVGAIIGKKGQHIKQLSRFASASIKIAPPETPDS<br>KVRMVIITGPPEAQFKAQGRIYGKLKEENFFGPKEEVKLETHIRVPASAAG<br>RVIGKGGKTVNELQNLTAAEVVVPRDQTPDENDQVIVKIIGHFYASQMAQR<br>KIRDILAQVKQQHQKGQSNQAQARRK
Full-length S181E	GPLGSPGIPGMNKLYIGNLNESVTPADLEKVFAEHKISYSGQFLVKSGYAF<br>VDCPDEHWAMKAIETFSGKVELQGKRLEIEHSVPKKQRSRKIQIRNIPPQL<br>RWEVLDSLLAQYGTVENCEQVNTESETAVVNVTYSNREQTRQAIMKLNG<br>HQLENHALKVSYIPDEQIAQGPENGRRGGFGSRGQPRQGEPVAAGAPAK<br>QQQVDIPLRLLVPTQYVGAIIGKEGATIRNITKQTQSKIDVHRKENAGAAEK<br>AISVHSTPEGCSSACKMILEIMHKEAKDTKTADEVPLKILAHNNFVGRLIGK<br>EGRNLKKVEQDTETKITISSLQDLTLYNPERTITVKGAIENCCRAEQEIMKK<br>VREAYENDVAAMSLQSHLIPGLNLAAVGLFPASSSAVPPPPSSVTGAAPYS<br>SFMQAPEQEMVQVFIPAQAVGAIIGKKGQHIKQLSRFASASIKIAPPETPDS<br>KVRMVIITGPPEAQFKAQGRIYGKLKEENFFGPKEEVKLETHIRVPASAAG<br>RVIGKGGKTVNELQNLTAAEVVVPRDQTPDENDQVIVKIIGHFYASQMAQR<br>KIRDILAQVKQQHQKGQSNQAQARRK
Full-length Y396E	GPLGSPGIPGMNKLYIGNLNESVTPADLEKVFAEHKISYSGQFLVKSGYAF<br>VDCPDEHWAMKAIETFSGKVELQGKRLEIEHSVPKKQRSRKIQIRNIPPQL<br>RWEVLDSLLAQYGTVENCEQVNTESETAVVNVTYSNREQTRQAIMKLNG<br>HQLENHALKVSYIPDEQIAQGPENGRRGGFGSRGQPRQGSPVAAGAPAK<br>QQQVDIPLRLLVPTQYVGAIIGKEGATIRNITKQTQSKIDVHRKENAGAAEK<br>AISVHSTPEGCSSACKMILEIMHKEAKDTKTADEVPLKILAHNNFVGRLIGK<br>EGRNLKKVEQDTETKITISSLQDLTLYNPERTITVKGAIENCCRAEQEIMKK<br>VREAYENDVAAMSLQSHLIPGLNLAAVGLFPASSSAVPPPPSSVTGAAPES<br>SFMQAPEQEMVQVFIPAQAVGAIIGKKGQHIKQLSRFASASIKIAPPETPDS<br>KVRMVIITGPPEAQFKAQGRIYGKLKEENFFGPKEEVKLETHIRVPASAAG<br>RVIGKGGKTVNELQNLTAAEVVVPRDQTPDENDQVIVKIIGHFYASQMAQR<br>KIRDILAQVKQQHQKGQSNQAQARRK
Full-length RQ	GPLGSPGIPGMNKLYIGNLNESVTPADLEKVFAEHKISYSGQFLVKSGYAF<br>VDCPDEHWAMKAIETFSGKVELQGKRLEIEHSVPKKQRSRKIQIRNIPPQL<br>RWEVLDSLLAQYGTVENCEQVNTESETAVVNVTYSNREQTRQAIMKLNG<br>HQLENHALKVSYIPDEQIAQGPENGQQGGFGSQGQPQQGSPVAAGAPA<br>KQQQVDIPLRLLVPTQYVGAIIGKEGATIRNITKQTQSKIDVHRKENAGAAE<br>KAISVHSTPEGCSSACKMILEIMHKEAKDTKTADEVPLKILAHNNFVGRLIG<br>KEGRNLKKVEQDTETKITISSLQDLTLYNPERTITVKGAIENCCRAEQEIMK<br>KVREAYENDVAAMSLQSHLIPGLNLAAVGLFPASSSAVPPPPSSVTGAAPY<br>SSFMQAPEQEMVQVFIPAQAVGAIIGKKGQHIKQLSRFASASIKIAPPETPD<br>SKVRMVIITGPPEAQFKAQGRIYGKLKEENFFGPKEEVKLETHIRVPASAA<br>GRVIGKGGKTVNELQNLTAAEVVVPRDQTPDENDQVIVKIIGHFYASQMAQ<br>RKIRDILAQVKQQHQKGQSNQAQARRK

mCherry-<br>IGF2BP1<br>full-length<br>wild-type	MVSKGEEDNMAIIKEFMRFKVHMEGSVNGHEFEIEGEGEGRPYEGTQTA<br>KLKVTKGGPLPFAWDILSPQFMYGSKAYVKHPADIPDYLKLSFPEGFKWE<br>RVMNFEDGGVVTVTQDSSLQDGEFIYKVKLRGTNFPSDGPVMQKKTMG<br>WEASSERMYPEDGALKGEIKQRLKLKDGGHYDAEVKTTYKAKKPVQLPG<br>AYNVNIKLDITSHNEDYTIVEQYERAEGRHSTGGMDELYKMNKLYIGNLNE<br>SVTPADLEKVFAEHKISYSGQFLVKSGYAFVDCPDEHWAMKAIETFSGKV<br>ELQGKRLEIEHSVPKKQRSRKIQIRNIPPQLRWEVLDSLLAQYGTVENCEQ<br>VNTESETAVVNVTYSNREQTRQAIMKLNGHQLENHALKVSYIPDEQIAQG<br>PENGRRGGFGSRGQPRQGSPVAAGAPAKQQQVDIPLRLLVPTQYVGAIIG<br>KEGATIRNITKQTQSKIDVHRKENAGAAEKAISVHSTPEGCSSACKMILEIM<br>HKEAKDTKTADEVPLKILAHNNFVGRLIGKEGRNLKKVEQDTETKITISSLQ<br>DLTLYNPERTITVKGAIENCCRAEQEIMKKVREAYENDVAAMSLQSHLIPGL<br>NLAAVGLFPASSSAVPPPPSSVTGAAPYSSFMQAPEQEMVQVFIPAQAVG<br>AIIGKKGQHIKQLSRFASASIKIAPPETPDSKVRMVIITGPPEAQFKAQGRIY<br>GKLKEENFFGPKEEVKLETHIRVPASAAGRVIGKGGKTVNELQNLTAAEVV<br>VPRDQTPDENDQVIVKIIGHFYASQMAQRKIRDILAQVKQQHQKGQSNQA<br>QARRKDLEVLFQ
mCherry-<br>IGF2BP1<br>full-length<br>S181E	MVSKGEEDNMAIIKEFMRFKVHMEGSVNGHEFEIEGEGEGRPYEGTQTA<br>KLKVTKGGPLPFAWDILSPQFMYGSKAYVKHPADIPDYLKLSFPEGFKWE<br>RVMNFEDGGVVTVTQDSSLQDGEFIYKVKLRGTNFPSDGPVMQKKTMG<br>WEASSERMYPEDGALKGEIKQRLKLKDGGHYDAEVKTTYKAKKPVQLPG<br>AYNVNIKLDITSHNEDYTIVEQYERAEGRHSTGGMDELYKMNKLYIGNLNE<br>SVTPADLEKVFAEHKISYSGQFLVKSGYAFVDCPDEHWAMKAIETFSGKV<br>ELQGKRLEIEHSVPKKQRSRKIQIRNIPPQLRWEVLDSLLAQYGTVENCEQ<br>VNTESETAVVNVTYSNREQTRQAIMKLNGHQLENHALKVSYIPDEQIAQG<br>PENGRRGGFGSRGQPRQGEPVAAGAPAKQQQVDIPLRLLVPTQYVGAIIG<br>KEGATIRNITKQTQSKIDVHRKENAGAAEKAISVHSTPEGCSSACKMILEIM<br>HKEAKDTKTADEVPLKILAHNNFVGRLIGKEGRNLKKVEQDTETKITISSLQ<br>DLTLYNPERTITVKGAIENCCRAEQEIMKKVREAYENDVAAMSLQSHLIPGL<br>NLAAVGLFPASSSAVPPPPSSVTGAAPYSSFMQAPEQEMVQVFIPAQAVG<br>AIIGKKGQHIKQLSRFASASIKIAPPETPDSKVRMVIITGPPEAQFKAQGRIY<br>GKLKEENFFGPKEEVKLETHIRVPASAAGRVIGKGGKTVNELQNLTAAEVV<br>VPRDQTPDENDQVIVKIIGHFYASQMAQRKIRDILAQVKQQHQKGQSNQA<br>QARRKDLEVLFQ
mCherry-<br>IGF2BP1<br>full-length<br>Y396E	MVSKGEEDNMAIIKEFMRFKVHMEGSVNGHEFEIEGEGEGRPYEGTQTA<br>KLKVTKGGPLPFAWDILSPQFMYGSKAYVKHPADIPDYLKLSFPEGFKWE<br>RVMNFEDGGVVTVTQDSSLQDGEFIYKVKLRGTNFPSDGPVMQKKTMG<br>WEASSERMYPEDGALKGEIKQRLKLKDGGHYDAEVKTTYKAKKPVQLPG<br>AYNVNIKLDITSHNEDYTIVEQYERAEGRHSTGGMDELYKMNKLYIGNLNE<br>SVTPADLEKVFAEHKISYSGQFLVKSGYAFVDCPDEHWAMKAIETFSGKV<br>ELQGKRLEIEHSVPKKQRSRKIQIRNIPPQLRWEVLDSLLAQYGTVENCEQ<br>VNTESETAVVNVTYSNREQTRQAIMKLNGHQLENHALKVSYIPDEQIAQG<br>PENGRRGGFGSRGQPRQGSPVAAGAPAKQQQVDIPLRLLVPTQYVGAIIG<br>KEGATIRNITKQTQSKIDVHRKENAGAAEKAISVHSTPEGCSSACKMILEIM<br>HKEAKDTKTADEVPLKILAHNNFVGRLIGKEGRNLKKVEQDTETKITISSLQ<br>DLTLYNPERTITVKGAIENCCRAEQEIMKKVREAYENDVAAMSLQSHLIPGL<br>NLAAVGLFPASSSAVPPPPSSVTGAAPESSFMQAPEQEMVQVFIPAQAVG<br>AIIGKKGQHIKQLSRFASASIKIAPPETPDSKVRMVIITGPPEAQFKAQGRIY

	GKLKEENFFGPKEEVKLETHIRVPASAAGRVIGKGGKTVNELQNLTAAEVV VPRDQTPDENDQVIVKIIGHFYASQMAQRKIRDILAQVKQQHQKGQSNQA QARRKDLEVLFQ
KH1-4 wild-type	GPGVDIPLRLLVPTQYVGAIIGKEGATIRNITKQTQSKIDVHRKENAGAAEK AISVHSTPEGCSSACKMILEIMHKEAKDTKTADEVPLKILAHNNFVGRLIGK EGRNLKKVEQDTETKITISSLQDLTLYNPERTITVKGAIENCCRAEQEIMKK VREAYENDVAAMSLQSHLIPGLNLAAVGLFPASSSAVPPPPSSVTGAAPYS SFMQAPEQEMVQVFIPAQAVGAIIGKKGQHIKQLSRFASASIKIAPPETPDS KVRMVIITGPPEAQFKAQGRIYGKLKEENFFGPKEEVKLETHIRVPASAAG RVIGKGGKTVNELQNLTAAEVVVPRDQTPDENDQVIVKIIGHFYASQMAQR KIRDILAQVKQQHQKGQSNQAQARRK
KH1-4 Y396E	GPGVDIPLRLLVPTQYVGAIIGKEGATIRNITKQTQSKIDVHRKENAGAAEK AISVHSTPEGCSSACKMILEIMHKEAKDTKTADEVPLKILAHNNFVGRLIGK EGRNLKKVEQDTETKITISSLQDLTLYNPERTITVKGAIENCCRAEQEIMKK VREAYENDVAAMSLQSHLIPGLNLAAVGLFPASSSAVPPPPSSVTGAAPES SFMQAPEQEMVQVFIPAQAVGAIIGKKGQHIKQLSRFASASIKIAPPETPDS KVRMVIITGPPEAQFKAQGRIYGKLKEENFFGPKEEVKLETHIRVPASAAG RVIGKGGKTVNELQNLTAAEVVVPRDQTPDENDQVIVKIIGHFYASQMAQR KIRDILAQVKQQHQKGQSNQAQARRK
RRM1-2 wild type	GPGMNKLYIGNLNESVTPADLEKVFAEHKISYSGQFLVKSGYAFVDCPDEH WAMKAIETFSGKVELQGKRLEIEHSVPKKQRSRKIQIRNIPPQLRWEVLDS LLAQYGTVENCEQVNTESETAVVNVTYSNREQTRQAIMKLNGHQLENHAL KVSYIPDEQIAQ
KH1-2 wild-type	GPGVDIPLRLLVPTQYVGAIIGKEGATIRNITKQTQSKIDVHRKENAGAAEK AISVHSTPEGCSSACKMILEIMHKEAKDTKTADEVPLKILAHNNFVGRLIGK EGRNLKKVEQDTETKITISSLQDLTLYNPERTITVKGAIENCCRAEQEIMKK VREAYENDVAAMSLQSHLIPGLN
KH3-4 wild-type	GPGEQEMVQVFIPAQAVGAIIGKKGQHIKQLSRFASASIKIAPPETPDSKVR MVIITGPPEAQFKAQGRIYGKLKEENFFGPKEEVKLETHIRVPASAAGRVIG KGGKTVNELQNLTAAEVVVPRDQTPDENDQVIVKIIGHFYASQMAQRKIRD ILAQVKQQHQKGQSNQAQARRK
L2-KH3-4 wild-type	GPGMKKVREAYENDVAAMSLQSHLIPGLNLAAVGLFPASSSAVPPPPSSV TGAAPYSSFMQAPEQEMVQVFIPAQAVGAIIGKKGQHIKQLSRFASASIKIA PPETPDSKVRMVIITGPPEAQFKAQGRIYGKLKEENFFGPKEEVKLETHIR VPASAAGRVIGKGGKTVNELQNLTAAEVVVPRDQTPDENDQVIVKIIGHFY ASQMAQRKIRDILAQVKQQHQKGQSNQAQARRK
Full-length KH3 GEEG KH4 GEEG	GPLGSPGIPGMNKLYIGNLNESVTPADLEKVFAEHKISYSGQFLVKSGYAF VDCPDEHWAMKAIETFSGKVELQGKRLEIEHSVPKKQRSRKIQIRNIPPQL RWEVLDSLLAQYGTVENCEQVNTESETAVVNVTYSNREQTRQAIMKLNG HQLENHALKVSYIPDEQIAQGPENGRRGGFGSRGQPRQGSPVAAGAPAK QQQVDIPLRLLVPTQYVGAIIGKEGATIRNITKQTQSKIDVHRKENAGAAEK AISVHSTPEGCSSACKMILEIMHKEAKDTKTADEVPLKILAHNNFVGRLIGK EGRNLKKVEQDTETKITISSLQDLTLYNPERTITVKGAIENCCRAEQEIMKK VREAYENDVAAMSLQSHLIPGLNLAAVGLFPASSSAVPPPPSSVTGAAPYS SFMQAPEQEMVQVFIPAQAVGAIIGEEGQHIKQLSRFASASIKIAPPETPDS KVRMVIITGPPEAQFKAQGRIYGKLKEENFFGPKEEVKLETHIRVPASAAG RVIGEEGKTVNELQNLTAAEVVVPRDQTPDENDQVIVKIIGHFYASQMAQR KIRDILAQVKQQHQKGQSNQAQARRK

KH3-4 KH3 wild-type KH4 GEEG	GPGEQEMVQVFIPAQAVGAIIGKKGQHIKQLSRFASASIKIAPPETPDSKVR MVIITGPPEAQFKAQGRIYGKLKEENFFGPKEEVKLETHIRVPASAAGRVIG EEGKTVNELQNLTAAEVVVPRDQTPDENDQVIVKIIGHFYASQMAQRKIRD ILAQVKQQHQKGQSNQAQARRK
KH3 GEEG KH4 wild-type	GPGEQEMVQVFIPAQAVGAIIGEEGQHIKQLSRFASASIKIAPPETPDSKVR MVIITGPPEAQFKAQGRIYGKLKEENFFGPKEEVKLETHIRVPASAAGRVIG KGGKTVNELQNLTAAEVVVPRDQTPDENDQVIVKIIGHFYASQMAQRKIRD ILAQVKQQHQKGQSNQAQARRK
Linker1 wild-type	ATYEQIAQGPENGRRGGFGSRGQPRQGSPVAAGAPAKQQQV
Linker1 S181E	ATYEQIAQGPENGRRGGFGSRGQPRQGEPVAAGAPAKQQQV
Linker1 RQ	ATYEQIAQGPENGQQGGFGSQGQPQQGSPVAAGAPAKQQQV
Linker2 wild-type	ATYMKKVREAYENDVAAMSLQSHLIPGLNLAAVGLFPASSSAVPPPPSSVT GAAPYSSFMQAPE
Linker2 Y396E	ATYMKKVREAYENDVAAMSLQSHLIPGLNLAAVGLFPASSSAVPPPPSSVT GAAPESSFMQAPE

Supplementary Table 9: Amino acid sequences of all recombinant IGF2BP1 protein constructs used in this study.

Supplementary References

1. Gruber, A.R. *et al.* The Vienna RNA Websuite. Nucleic Acids Res., vol. 36, no. suppl_2, pp. W70–W74, Jul (2008).
2. Hafner, M. et al. Transcriptome-wide identification of RNA-binding protein and microRNA target sites by PAR-CLIP. Cell 141, 129-141 (2010).
3. Zhao, W. et al. POSTAR3: An updated platform for exploring post-transcriptional regulation coordinated by RNA-binding proteins. Nucleic Acids Res 50, D287–D294 (2022).
4. Supek, F., Bošnjak, M., Škunca, N. & Šmuc, T. REVIGO Summarizes and Visualizes Long Lists of Gene Ontology Terms. PLoS One 6, e21800 (2011).
5. Conway, A.E. *et al.* Enhanced CLIP Uncovers IMP Protein-RNA Targets in Human Pluripotent Stem Cells Important for Cell Adhesion and Survival. *Cell Rep* 15, 666-679 (2016).
6. Schneider, T. *et al.* Combinatorial recognition of clustered RNA elements by the multidomain RNA-binding protein IMP3. *Nat Commun* 10, 2266 (2019).
7. Hafner, M. *et al.* Transcriptome-wide identification of RNA-binding protein and microRNA target sites by PAR-CLIP. *Cell* 141, 129-141 (2010).
8. Anisimova A.S. and Karagöz G.E. Optimized infrared photoactivatable ribonucleoside-enhanced crosslinking and immunoprecipitation (IR-PAR-CLIP) protocol identifies novel IGF2BP3-interacting RNAs in colon cancer cells. RNA, vol. 29, no. 11, pp. 1818–1836 (2023).
9. Bernstein P.L. *et al.* Control of c-myc mRNA half-life in vitro by a protein capable of binding to a coding region stability determinant. Genes Dev vol. 6, no. 4, pp. 642–654 (1992).
10. Chao, J.A. *et al.* ZBP1 recognition of beta-actin zipcode induces RNA looping. *Genes Dev* 24, 148-158 (2010).
11. Hüttelmaier S. et al. Spatial regulation of β-actin translation by Src-dependent phosphorylation of ZBP1. Nature vol. 438 no. 7067, pp. 512–515 (2005).
