# Supplementary material for: IGF2BP1 phosphorylation in the disordered linkers regulates ribonucleoprotein condensate formation and RNA metabolism
Source: Nat Commun. 2024 Oct 20;15:9054. doi: 10.1038/s41467-024-53400-4 (PMC11490574; doi:10.1038/s41467-024-53400-4)
Supplement: Supplementary file 2 — Reporting Summary [file 41467_2024_53400_MOESM2_ESM.pdf]

Corresponding author(s): G Elif Karagöz

Last updated by author(s): Oct 3, 2024

Reporting Summary

Nature Portfolio wishes to improve the reproducibility of the work that we publish. This form provides structure for consistency and transparency in reporting. For further information on Nature Portfolio policies, see our Editorial Policies and the Editorial Policy Checklist.

Statistics

For all statistical analyses, confirm that the following items are present in the figure legend, table legend, main text, or Methods section.

n/a	Confirmed	
☐	☒	The exact sample size (*n*) for each experimental group/condition, given as a discrete number and unit of measurement
☐	☒	A statement on whether measurements were taken from distinct samples or whether the same sample was measured repeatedly
☐	☒	The statistical test(s) used AND whether they are one- or two-sided<br>*Only common tests should be described solely by name; describe more complex techniques in the Methods section.*
☒	☐	A description of all covariates tested
☐	☒	A description of any assumptions or corrections, such as tests of normality and adjustment for multiple comparisons
☐	☒	A full description of the statistical parameters including central tendency (e.g. means) or other basic estimates (e.g. regression coefficient) AND variation (e.g. standard deviation) or associated estimates of uncertainty (e.g. confidence intervals)
☐	☒	For null hypothesis testing, the test statistic (e.g. *F*, *t*, *r*) with confidence intervals, effect sizes, degrees of freedom and *P* value noted<br>*Give P values as exact values whenever suitable.*
☒	☐	For Bayesian analysis, information on the choice of priors and Markov chain Monte Carlo settings
☒	☐	For hierarchical and complex designs, identification of the appropriate level for tests and full reporting of outcomes
☒	☐	Estimates of effect sizes (e.g. Cohen's *d*, Pearson's *r*), indicating how they were calculated

Our web collection on statistics for biologists contains articles on many of the points above.

Software and code

Policy information about availability of computer code

Data collection	Software used for data collection is stated in figure legends and/or materials and methods. Brightfield images for RNP granule formation assays and immunofluorescence images were acquired using Zen 3.3 blue edition. FRAP experiments very performed in VisiView 6.0. NMR data was acquired with Bruker TopSpin 3.5. Fluorescence Anisotropy experiments very carried out on a Edinburgh Instruments FS5 spectrofluorometer using the manufacturer provided software. The turbidity experiment was performed on a BioTek Synergy H1 plate reader with the manufacturers software. DLS data was recored by using DYNAMICS V7 from Wyatt.
Data analysis	Software used for data analysis is stated in figure legends and/or materials and methods. NMR data was processed with NMRPipe and analyzed with CcpNmr V3. Chemical Shift Perturbations and intensity ratios from NMR data were calculated with Excel from Microsoft Office Professional Plus 2016. GraphPad Prism 10 was used for all statistical tests and curve fittings. Image analysis and quantification was performed using Fiji/ImageJ 1.53t. Analysis of RNA-Seq and RIP-Seq was performed using bcl2fastq v2.20.0.422, fastqc 0.11.9, STAR v2.7.11b , Subread package v2.0.6 and edgeR package in R 4.4.1. DLS data was analysed by using DYNAMICS V7 from Wyatt, the Raynals online tool (https://spc.embl-hamburg.de/app/raynals) and GraphPad Prism 10.

For manuscripts utilizing custom algorithms or software that are central to the research but not yet described in published literature, software must be made available to editors and reviewers. We strongly encourage code deposition in a community repository (e.g. GitHub). See the Nature Portfolio guidelines for submitting code & software for further information.

Data

Policy information about availability of data

All manuscripts must include a data availability statement. This statement should provide the following information, where applicable:

- Accession codes, unique identifiers, or web links for publicly available datasets
- A description of any restrictions on data availability
- For clinical datasets or third party data, please ensure that the statement adheres to our policy

The mass spectrometry proteomics data have been deposited to the ProteomeXchange Consortium (http://proteomecentral.proteomexchange.org) via the PRIDE partner repository (Perez-Riverol et al., 2021) with the dataset identifier PXD045761 (reviewer's account: reviewer_pxd045761@ebi.ac.uk, password: Eiv17kZM).The mass spectrometry data of ProAlanase digested samples have been deposited via the PRIDE partner repository. Dataset identifier: PXD056497 (reviewer's account: reviewer_pxd056497@ebi.ac.uk, password: S7lBO5DOUiKD). This data will be made publicly accessible after the paper is published.
The NMR signal assignments are deposited in the Biological Magnetic Resonance Data Base (BMRB) with the BMRB IDs of 52567 (linker1) and 52568 (linker2).
Sequencing data generated in this study have been deposited in the Gene Expression Omnibus database, https://www.ncbi.nlm.nih.gov/geo/ (accession no. GSE272875).

Research involving human participants, their data, or biological material

Policy information about studies with human participants or human data. See also policy information about sex, gender (identity/presentation), and sexual orientation and race, ethnicity and racism.

Reporting on sex and gender	*Use the terms sex (biological attribute) and gender (shaped by social and cultural circumstances) carefully in order to avoid confusing both terms. Indicate if findings apply to only one sex or gender; describe whether sex and gender were considered in study design; whether sex and/or gender was determined based on self-reporting or assigned and methods used.* *Provide in the source data disaggregated sex and gender data, where this information has been collected, and if consent has been obtained for sharing of individual-level data; provide overall numbers in this Reporting Summary. Please state if this information has not been collected.* *Report sex- and gender-based analyses where performed, justify reasons for lack of sex- and gender-based analysis.*
Reporting on race, ethnicity, or other socially relevant groupings	*Please specify the socially constructed or socially relevant categorization variable(s) used in your manuscript and explain why they were used. Please note that such variables should not be used as proxies for other socially constructed/relevant variables (for example, race or ethnicity should not be used as a proxy for socioeconomic status).* *Provide clear definitions of the relevant terms used, how they were provided (by the participants/respondents, the researchers, or third parties), and the method(s) used to classify people into the different categories (e.g. self-report, census or administrative data, social media data, etc.)* *Please provide details about how you controlled for confounding variables in your analyses.*
Population characteristics	*Describe the covariate-relevant population characteristics of the human research participants (e.g. age, genotypic information, past and current diagnosis and treatment categories). If you filled out the behavioural & social sciences study design questions and have nothing to add here, write "See above."*
Recruitment	*Describe how participants were recruited. Outline any potential self-selection bias or other biases that may be present and how these are likely to impact results.*
Ethics oversight	*Identify the organization(s) that approved the study protocol.*

Note that full information on the approval of the study protocol must also be provided in the manuscript.

Field-specific reporting

Please select the one below that is the best fit for your research. If you are not sure, read the appropriate sections before making your selection.

[x] Life sciences [ ] Behavioural & social sciences [ ] Ecological, evolutionary & environmental sciences

For a reference copy of the document with all sections, see nature.com/documents/nr-reporting-summary-flat.pdf

Life sciences study design

All studies must disclose on these points even when the disclosure is negative.

Sample size	Sample sizes where chosen based on published studies with similar designed experiments.
Data exclusions	Outliers in RNP granules quantification experiments were excluded using the ROUT method in GraphPad Prism using Q = 1%. Data points in turbidity assays that showed strong scattering at timepoint 0 (mostly contaminants in the light path) were excluded.
Replication	Sample sizes and replicates are stated in figure legends and/or material and methods. RNP granule formation assays without mCherry-labeled proteins were carried out in duplicates. EMSAs were performed duplicates. Fluorescence anisotropy was measured in triplicates. RNA-Seq and RIP-Seq were performed in triplicates.

Randomization	No randomization was used in experiments.
Blinding	No blinding was performed during experiments.

Reporting for specific materials, systems and methods

We require information from authors about some types of materials, experimental systems and methods used in many studies. Here, indicate whether each material, system or method listed is relevant to your study. If you are not sure if a list item applies to your research, read the appropriate section before selecting a response.

Materials & experimental systems

n/a	Involved in the study
☐	☒ Antibodies
☐	☒ Eukaryotic cell lines
☒	☐ Palaeontology and archaeology
☒	☐ Animals and other organisms
☒	☐ Clinical data
☒	☐ Dual use research of concern
☒	☐ Plants

Methods

n/a	Involved in the study
☒	☐ ChIP-seq
☒	☐ Flow cytometry
☒	☐ MRI-based neuroimaging

Antibodies

Antibodies used	Anti-IGF2BP1 Proteintech 22803-1-AP 00018571; Anti-GAPDH Proteintech 10494-1-AP 00113796; Anti-Rabbit IgG HRP conjugate Promega W401B 0000573275; Anti-Rabbit IgG IRdye 800CW secondary antibody LI-COR 926-32211 D30307-15;
Validation	Anti-IGF2BP1 antibody LotNr. 00018571 was validated for Western Blots in HEK293, HepG2 and Jurkat cells.<br>Anti-GAPDH antibody LotNr. 00113796 was validated for Western Blots in HEK293, HeLA, Jurkat, NIH/3T3 and C6 cells as well as in mouse brain and rat brain tissue.

Eukaryotic cell lines

Policy information about cell lines and Sex and Gender in Research

Cell line source(s)	HCT116 cells were obtained from the Masato Kanemaki lab (Natsume et al. 2016).<br>U2OS cells were received from Witold Szaflarski, Poznan University of Medical Sciences. Original source: Prof. Paul Anderson, Division of Rheumatology, Immunology and Allergy, Brigham and Women's Hospital, Boston, Massachusetts, USA. (https://doi.org/10.1016/S0076-6879(08)02626-8)
Authentication	None of the cell lines were not authenticated.
Mycoplasma contamination	HCT116 cells were tested for mycoplasma contamination and mycoplasma contamination was not detected.<br>U2OS cells were not tested for mycoplasma contamination.
Commonly misidentified lines (See ICLAC register)	*Name any commonly misidentified cell lines used in the study and provide a rationale for their use.*

Plants

Seed stocks	*Report on the source of all seed stocks or other plant material used. If applicable, state the seed stock centre and catalogue number. If plant specimens were collected from the field, describe the collection location, date and sampling procedures.*
Novel plant genotypes	*Describe the methods by which all novel plant genotypes were produced. This includes those generated by transgenic approaches, gene editing, chemical/radiation-based mutagenesis and hybridization. For transgenic lines, describe the transformation method, the number of independent lines analyzed and the generation upon which experiments were performed. For gene-edited lines, describe the editor used, the endogenous sequence targeted for editing, the targeting guide RNA sequence (if applicable) and how the editor was applied.*
Authentication	*Describe any authentication procedures for each seed stock used or novel genotype generated. Describe any experiments used to assess the effect of a mutation and, where applicable, how potential secondary effects (e.g. second site T-DNA insertions, mosiacism, off-target gene editing) were examined.*
