# Supplementary material for: IGF2BP1 phosphorylation in the disordered linkers regulates ribonucleoprotein condensate formation and RNA metabolism
Source: Nat Commun. 2024 Oct 20;15:9054. doi: 10.1038/s41467-024-53400-4 (PMC11490574; doi:10.1038/s41467-024-53400-4)
Supplement: Supplementary file 3 — Description of Additional Supplementary Files [file 41467_2024_53400_MOESM3_ESM.pdf]

Description of Additional Supplementary Files

File Name: SupplementaryData1

Description: MS data of *in vivo* and *in vitro* Pro-Alanase treated samples

File Name: SupplementaryData2

Description: FACS strategy and data to generate HCT116 cell lines for RNP granule quantification (Fig.3E-H)

File Name: SupplementaryData3

Description: FACS strategy and data to generate U2OS cell lines for RNP granule quantification (Fig.3I, Supp.Fig.6G-J)

File Name: SupplementaryData4

Description: FACS strategy and data to generate U2OS cell lines for RNA-Seq and RIP-Seq experiments (Fig.7A,B, Fig.Supp.12A-E)

File Name: SupplementaryData5

Description: Source data for RNA- and RIP-Seq of HCT116 cells expressing mCherry-IGF2BP1 wild-type and S181E, Y396E, and RQ mutants, and parental HCT116 cells in control and arsenate stress conditions
