# Supplementary figures and images for: IGF2BP1 phosphorylation in the disordered linkers regulates ribonucleoprotein condensate formation and RNA metabolism
Source: Nat Commun. 2024 Oct 20;15:9054. doi: 10.1038/s41467-024-53400-4 (PMC11490574; doi:10.1038/s41467-024-53400-4)

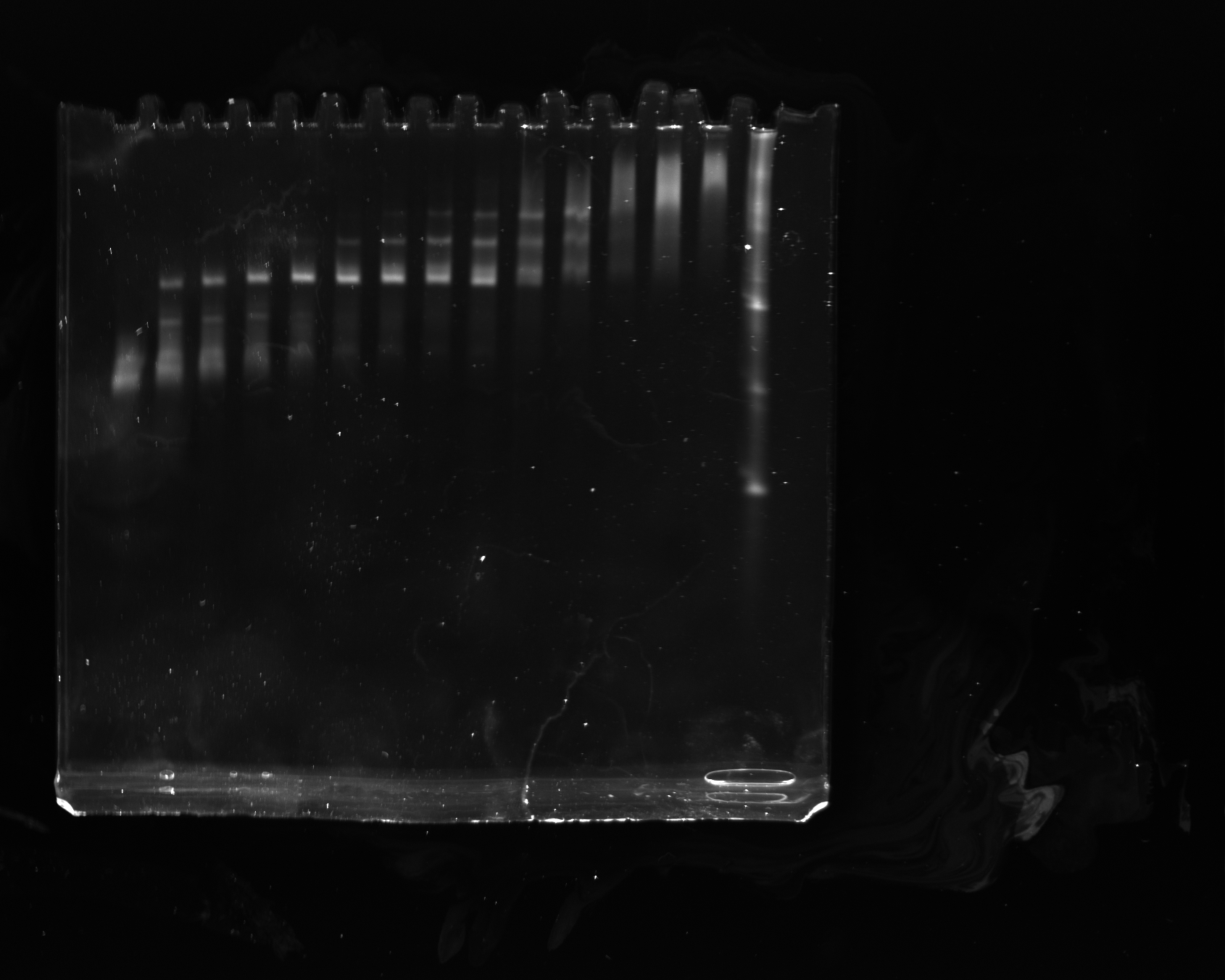

Supplement: Supplementary file 10 — Source Data [file 41467_2024_53400_MOESM10_ESM.zip › Figure 2A/Figure 2A_SupplementaryTable2_EMSA_XBP1_WT_A.tif]

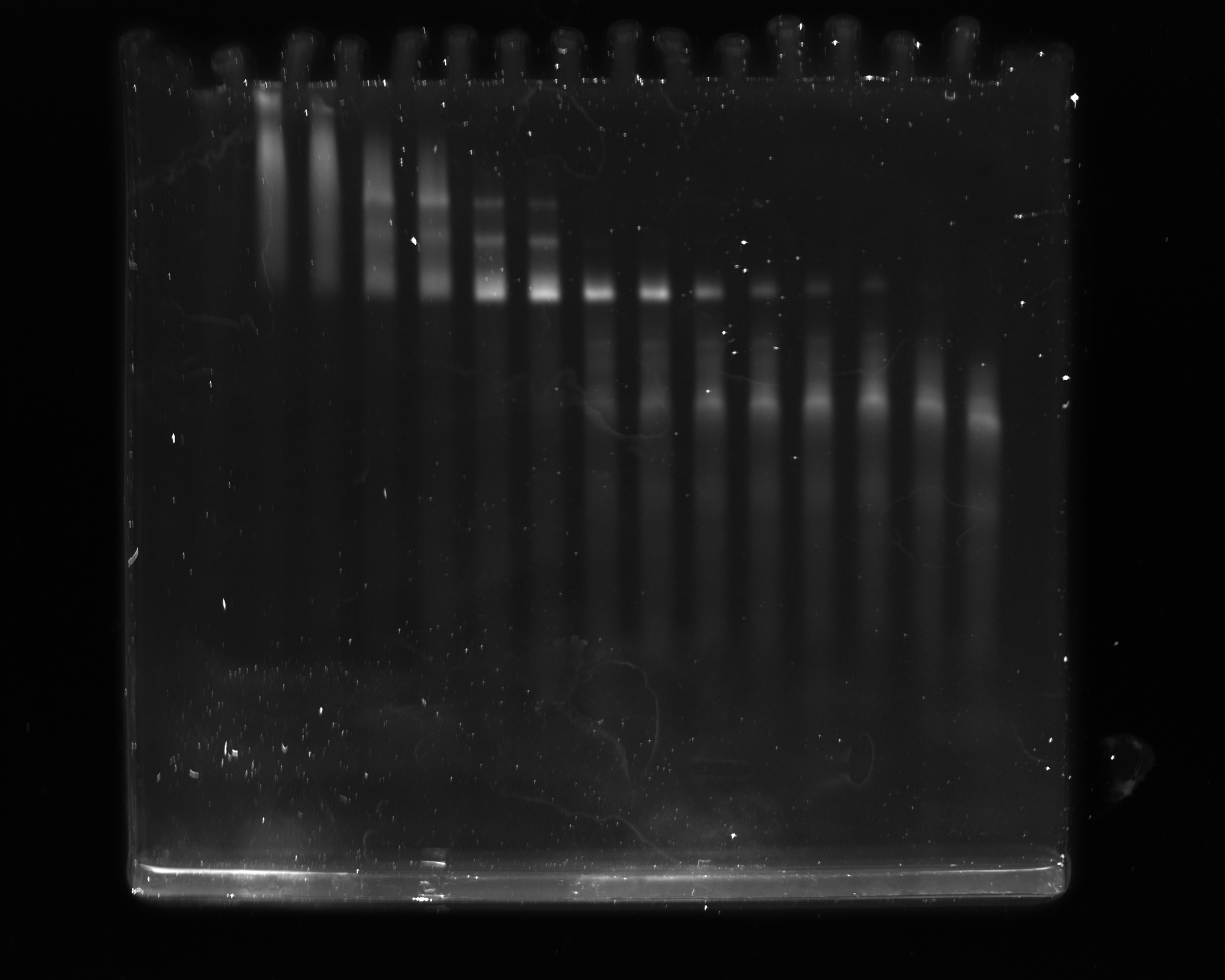

Supplement: Supplementary file 10 — Source Data [file 41467_2024_53400_MOESM10_ESM.zip › Figure 2A/SuppplementaryTable2_EMSA_XBP1_WT_B.tif]

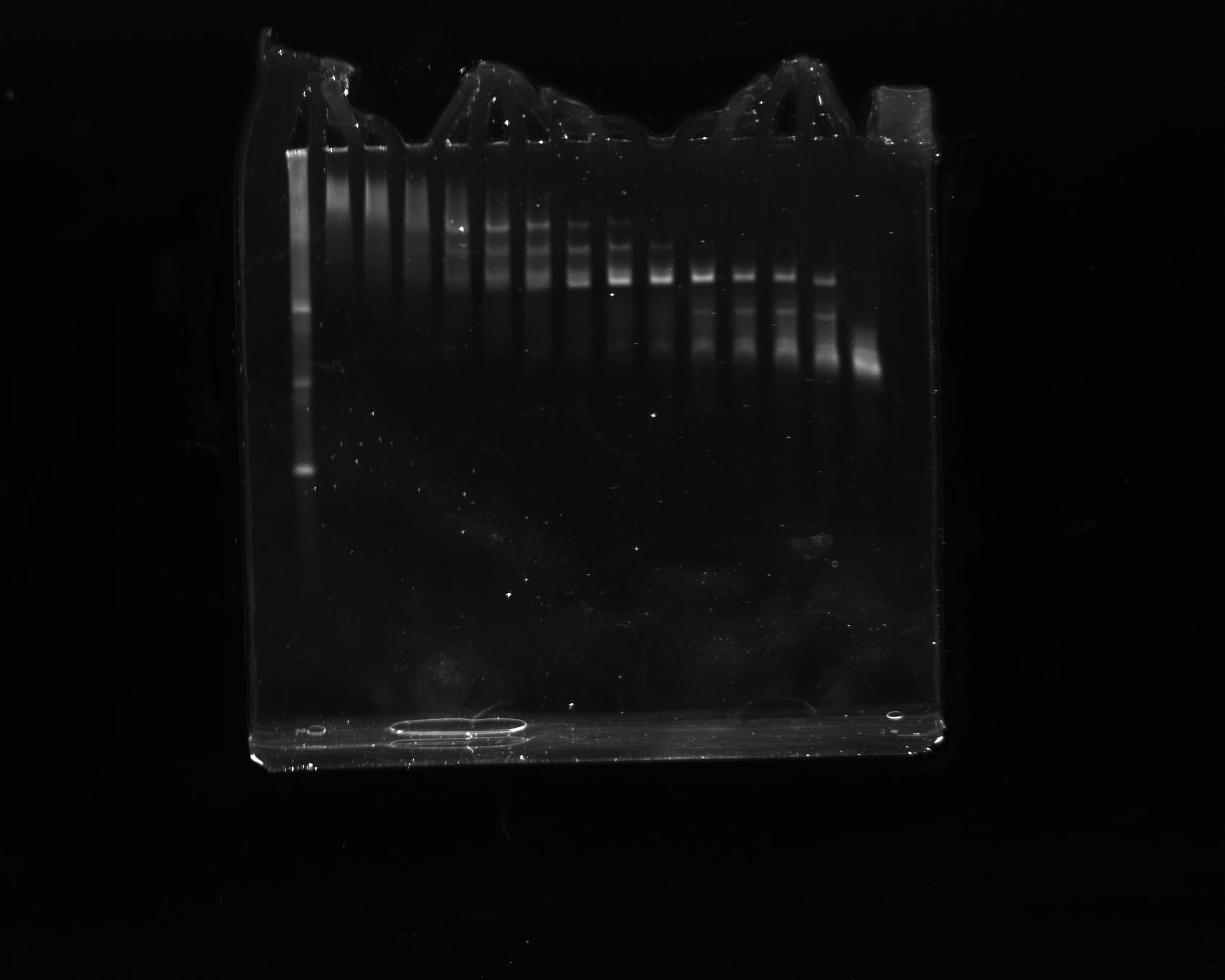

Supplement: Supplementary file 10 — Source Data [file 41467_2024_53400_MOESM10_ESM.zip › Figure 2B/Figure 2B_SupplementaryTable2_EMSA_XBP1_S181E_A.tif]

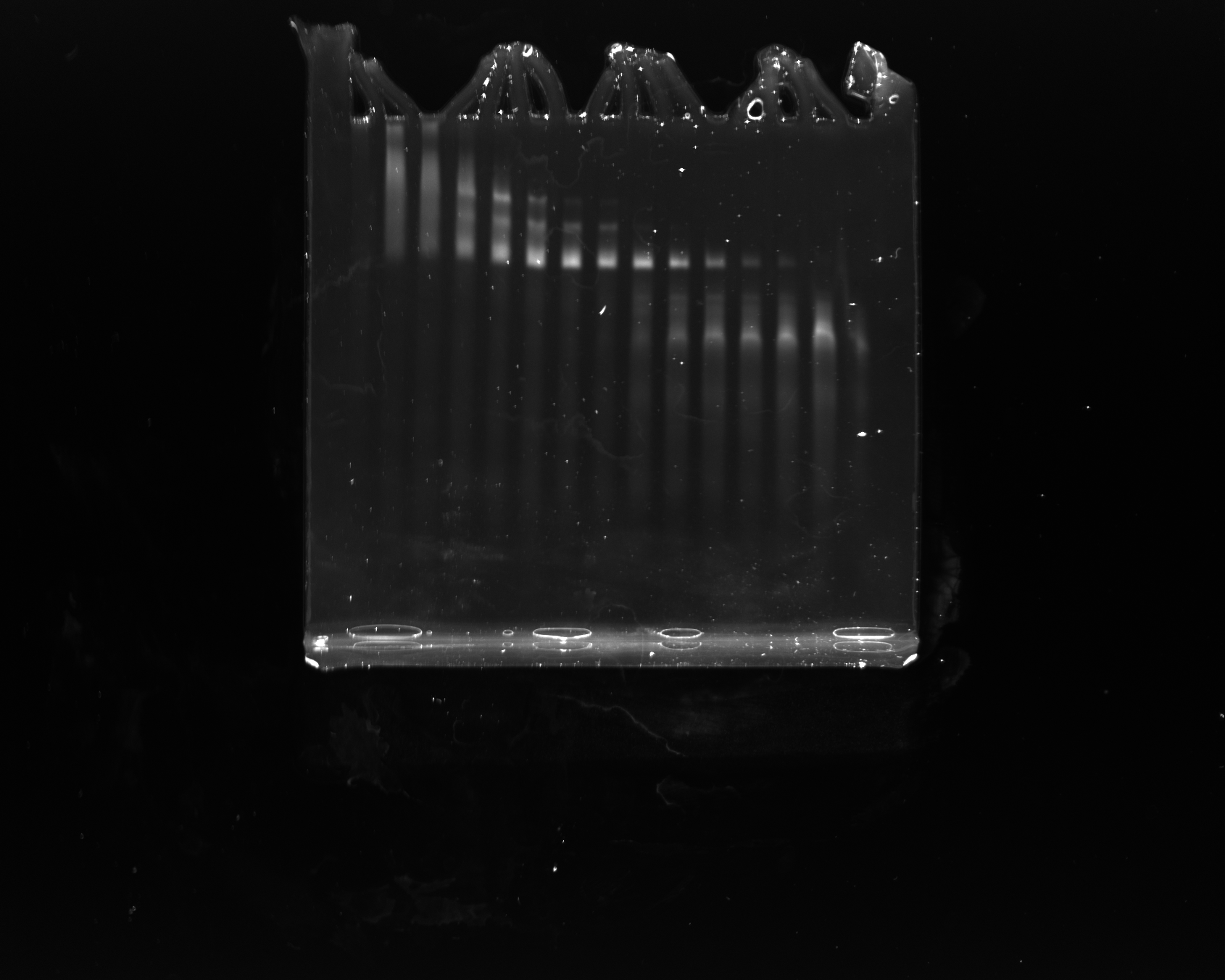

Supplement: Supplementary file 10 — Source Data [file 41467_2024_53400_MOESM10_ESM.zip › Figure 2B/SupplementaryTable2_EMSA_XBP1_S181E_B.tif]

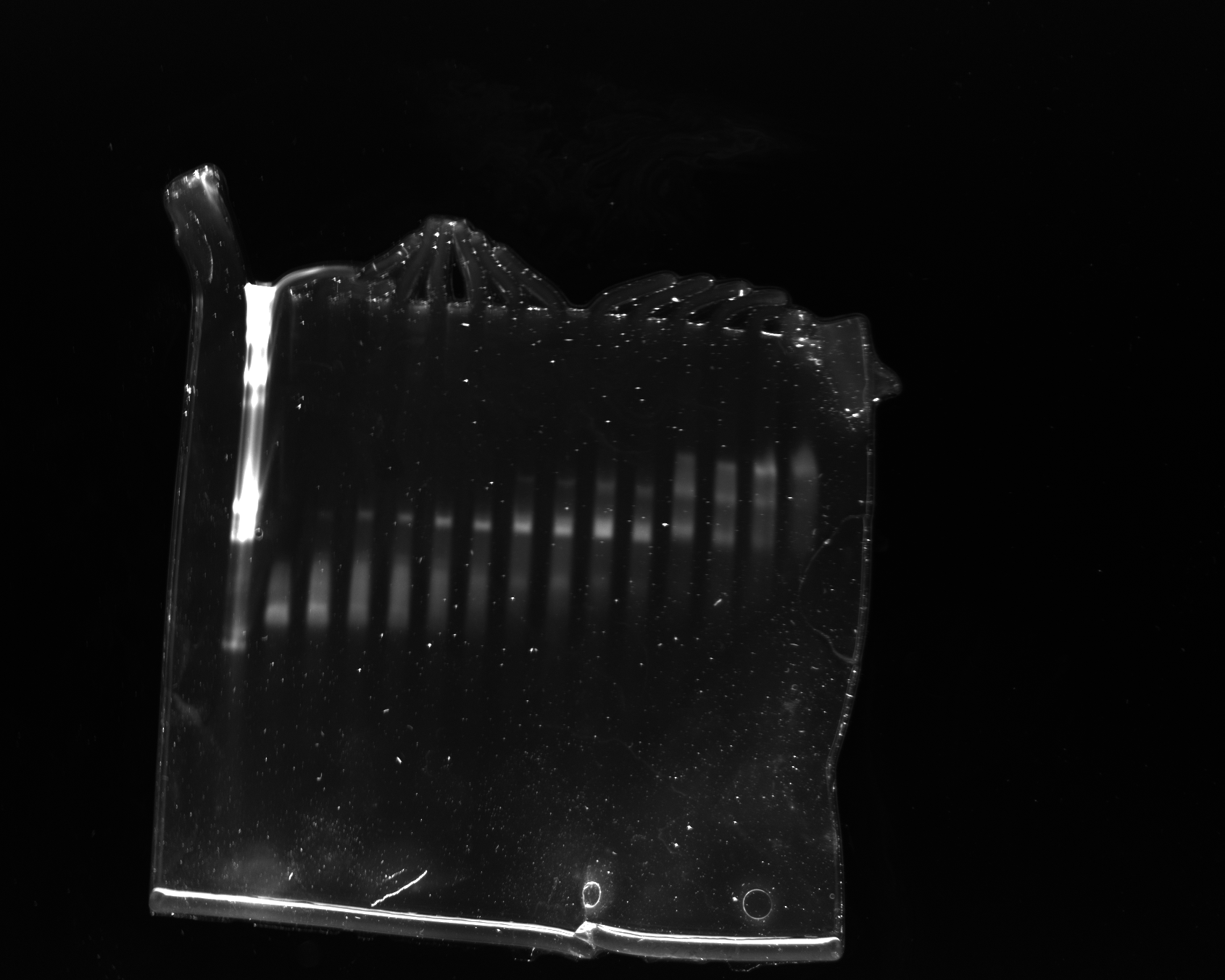

Supplement: Supplementary file 10 — Source Data [file 41467_2024_53400_MOESM10_ESM.zip › Figure 2C/Figure2C_SupplementaryTable2_EMSA_XBP1_Y396E_A.tif]

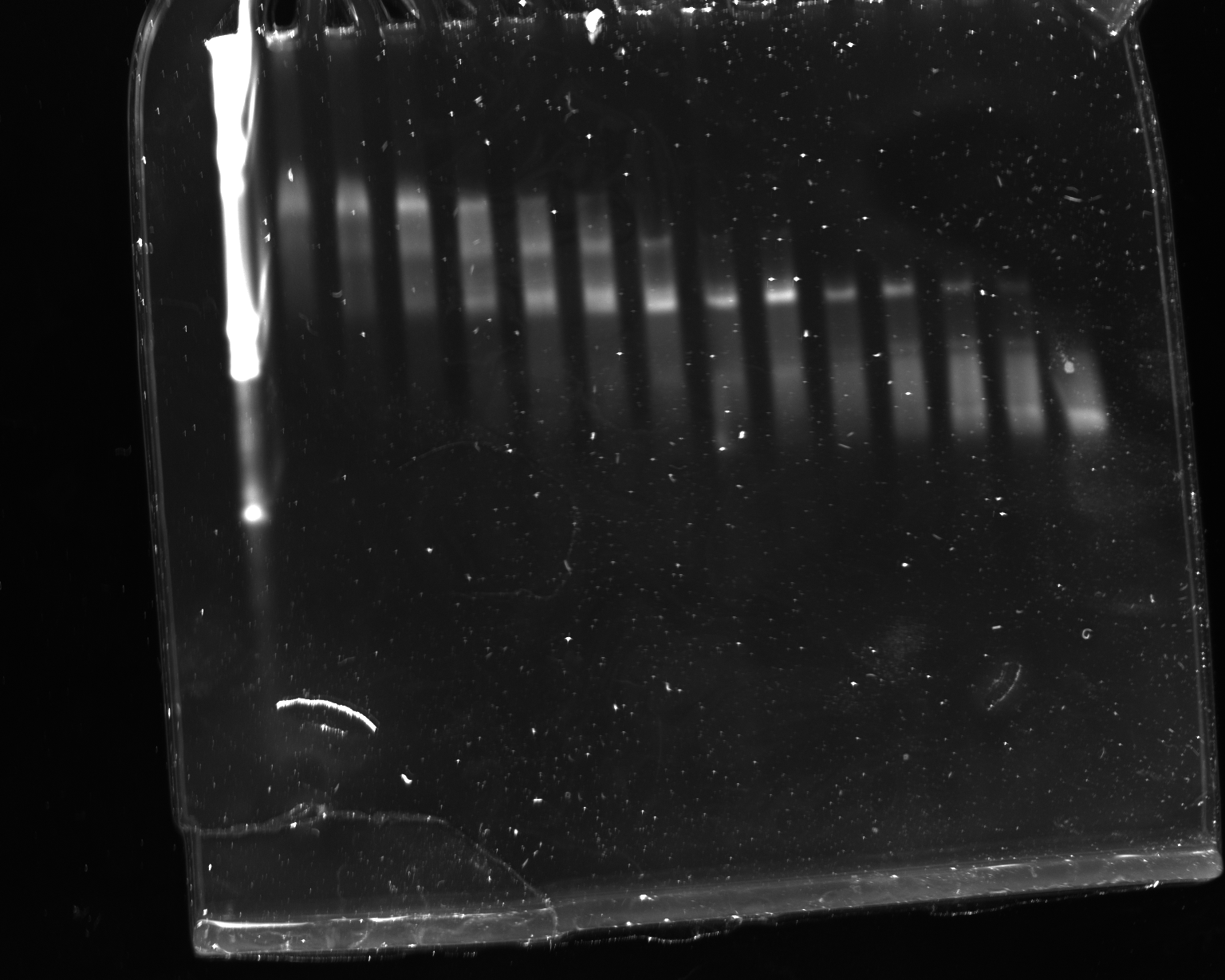

Supplement: Supplementary file 10 — Source Data [file 41467_2024_53400_MOESM10_ESM.zip › Figure 2C/SupplementaryTable2_EMSA_XBP1_Y396E_B.tif]

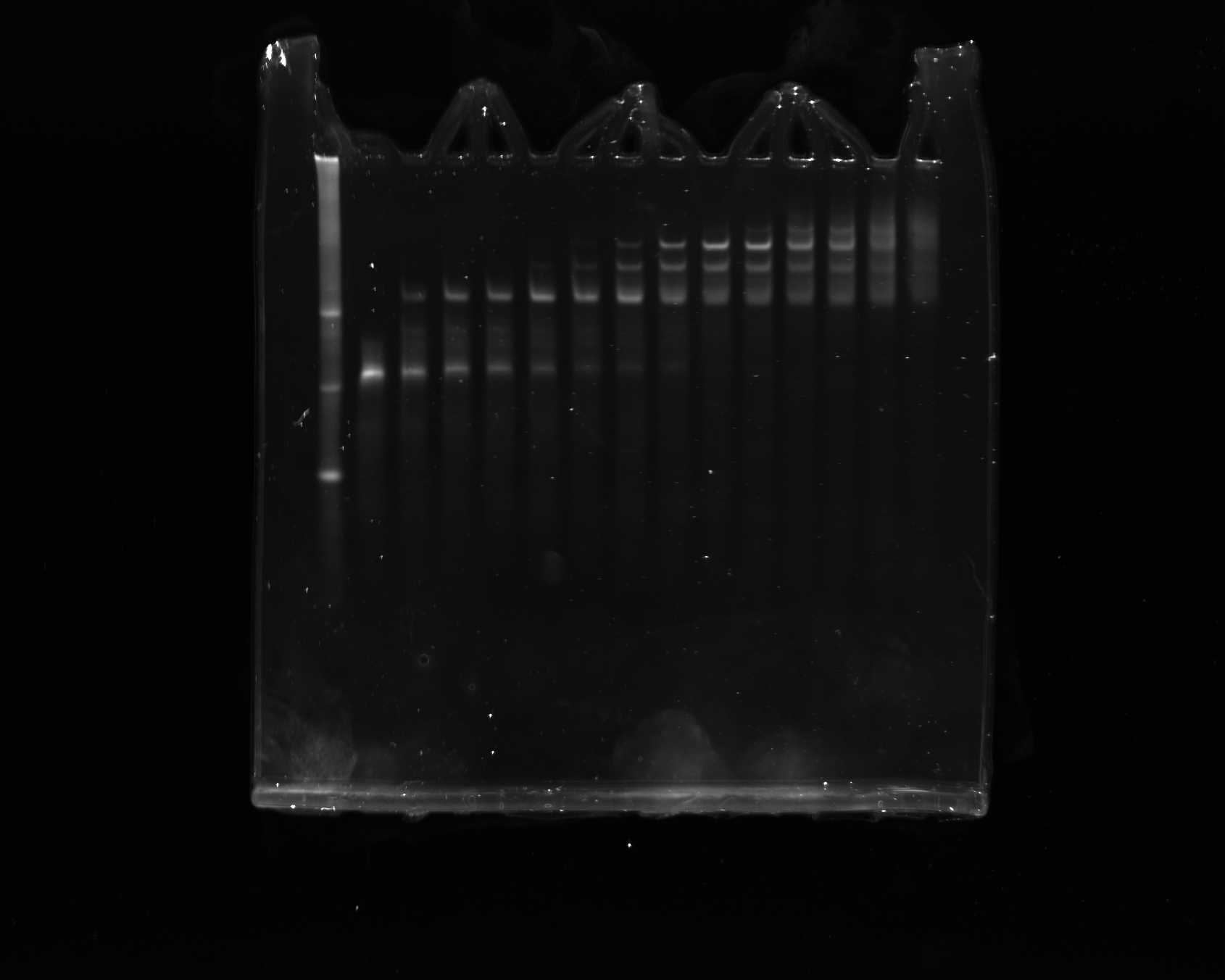

Supplement: Supplementary file 10 — Source Data [file 41467_2024_53400_MOESM10_ESM.zip › Supplementary Figure 11K/SupplementaryFigure11K_SupplementaryTable2_EMSA_XBP1_RQ_A.tif]

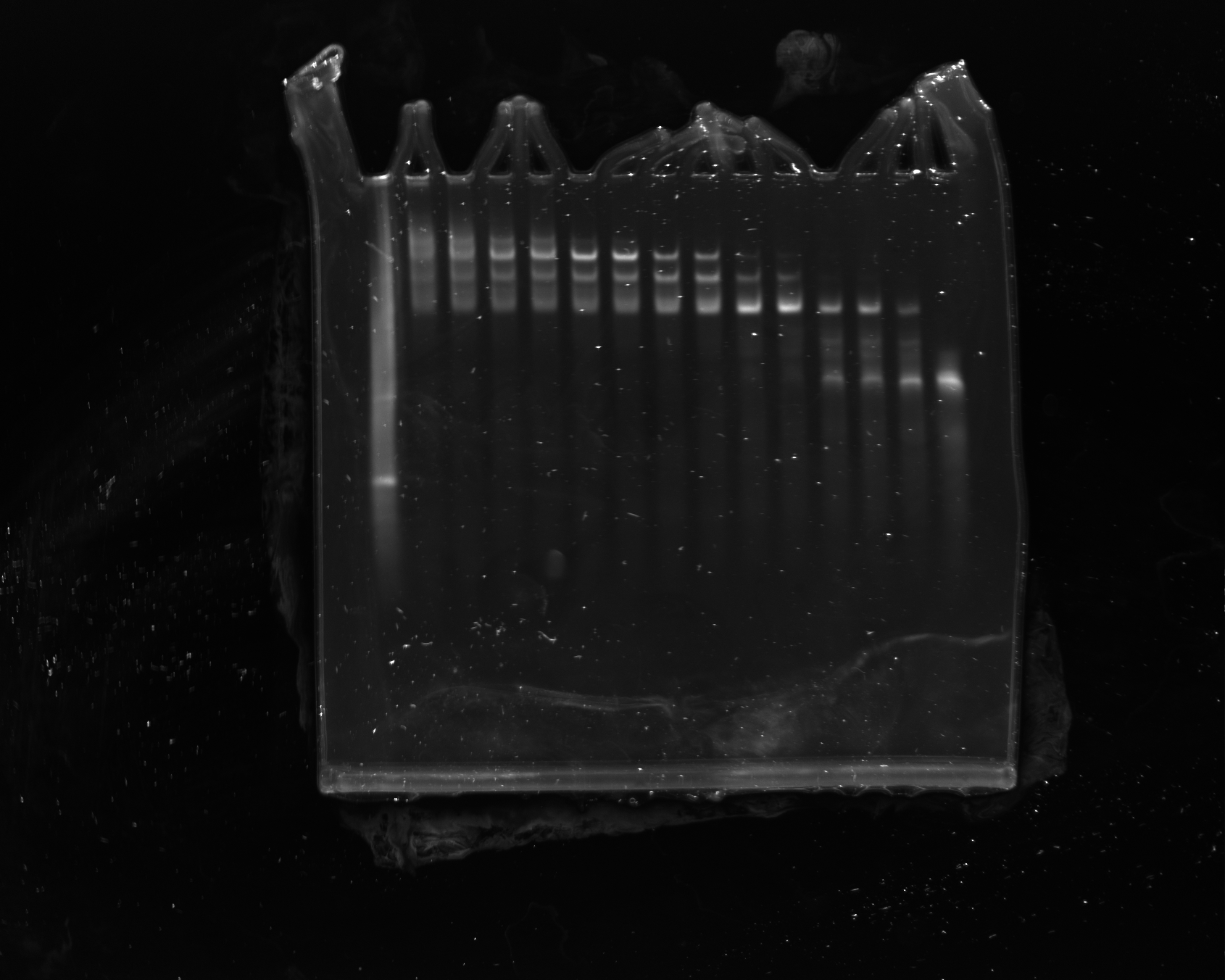

Supplement: Supplementary file 10 — Source Data [file 41467_2024_53400_MOESM10_ESM.zip › Supplementary Figure 11K/SupplementaryTable2_EMSA_XBP1_RQ_B.tif]

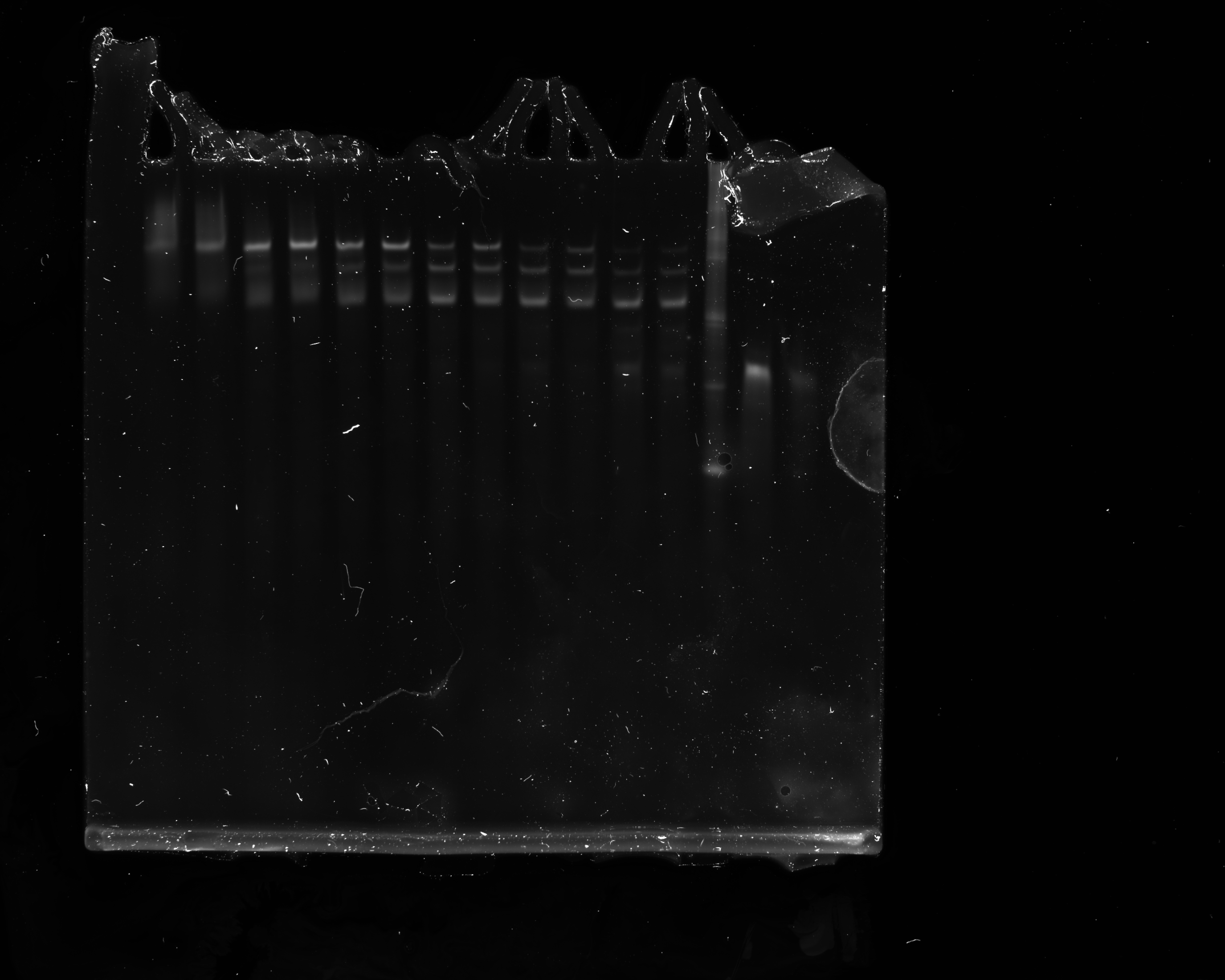

Supplement: Supplementary file 10 — Source Data [file 41467_2024_53400_MOESM10_ESM.zip › Supplementary Figure 2D/SupplementaryFigure2D_SupplementaryTable2_EMSA_EIF2A_WT_S181E_A.tif]

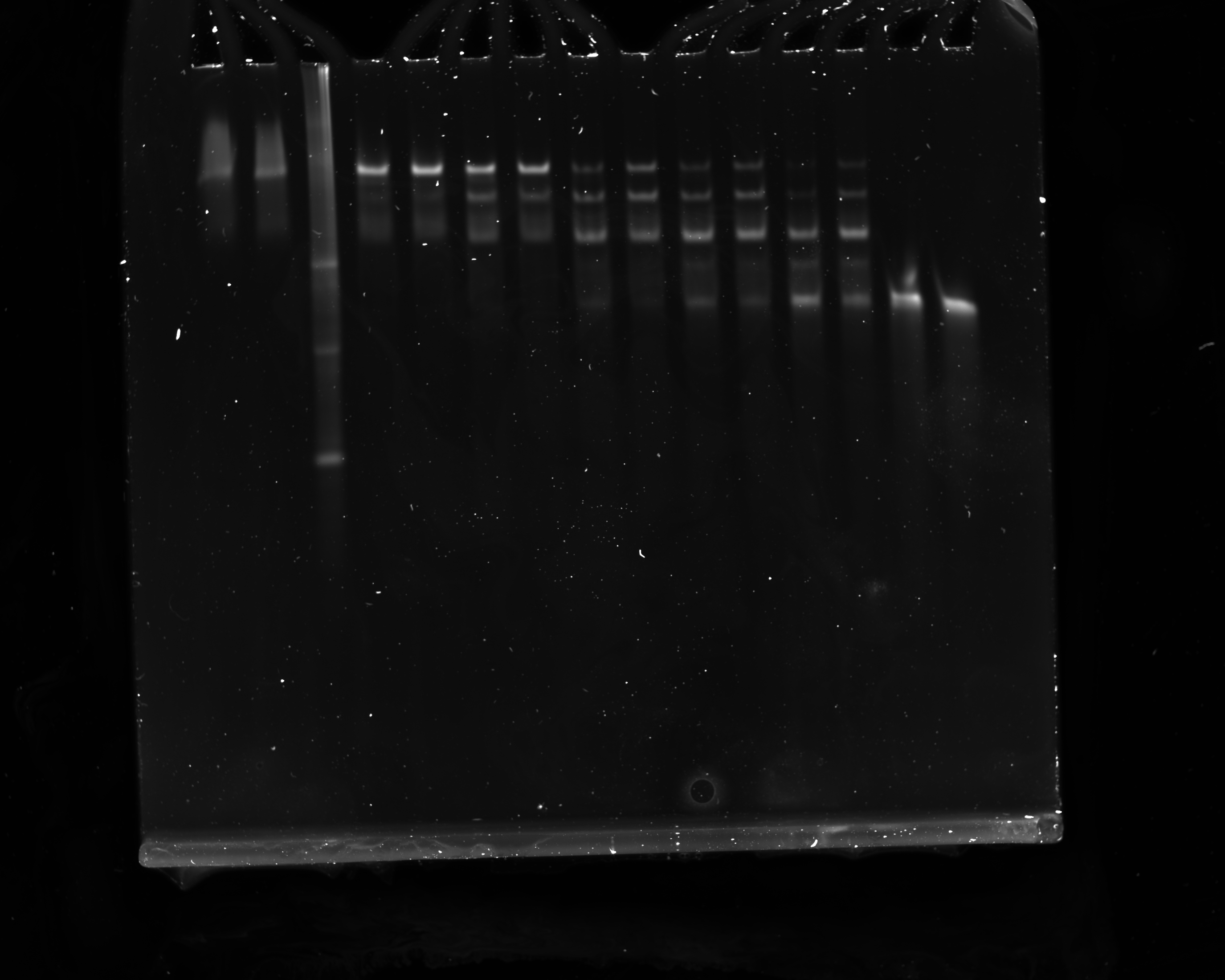

Supplement: Supplementary file 10 — Source Data [file 41467_2024_53400_MOESM10_ESM.zip › Supplementary Figure 2D/SupplementaryTable2_EMSA_EIF2A_WT_S181E_B.tif]

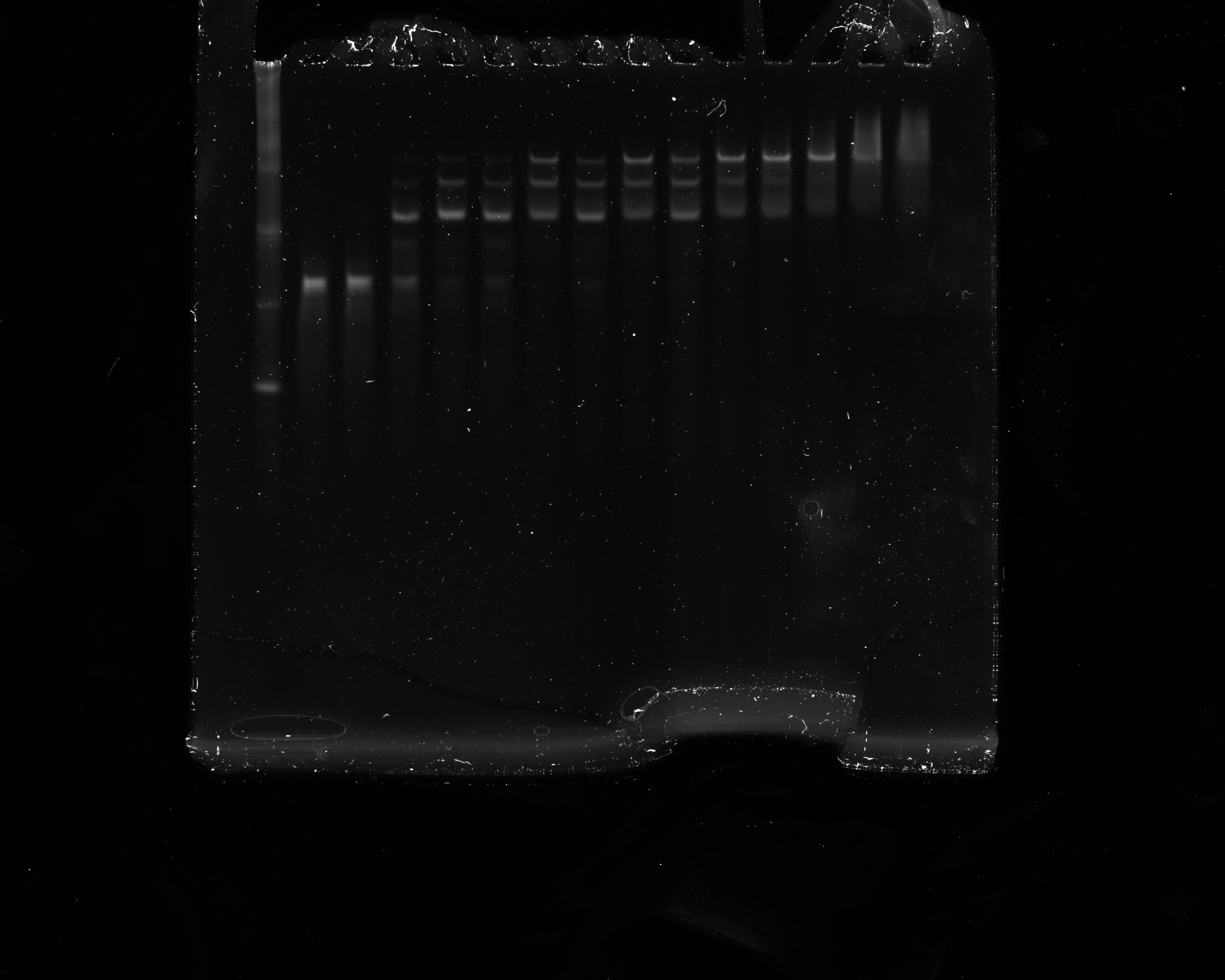

Supplement: Supplementary file 10 — Source Data [file 41467_2024_53400_MOESM10_ESM.zip › Supplementary Figure 2E/SupplementaryFigure2E_SupplementaryTable2_EMSA_EIF2A_WT_Y396E_A.tif]

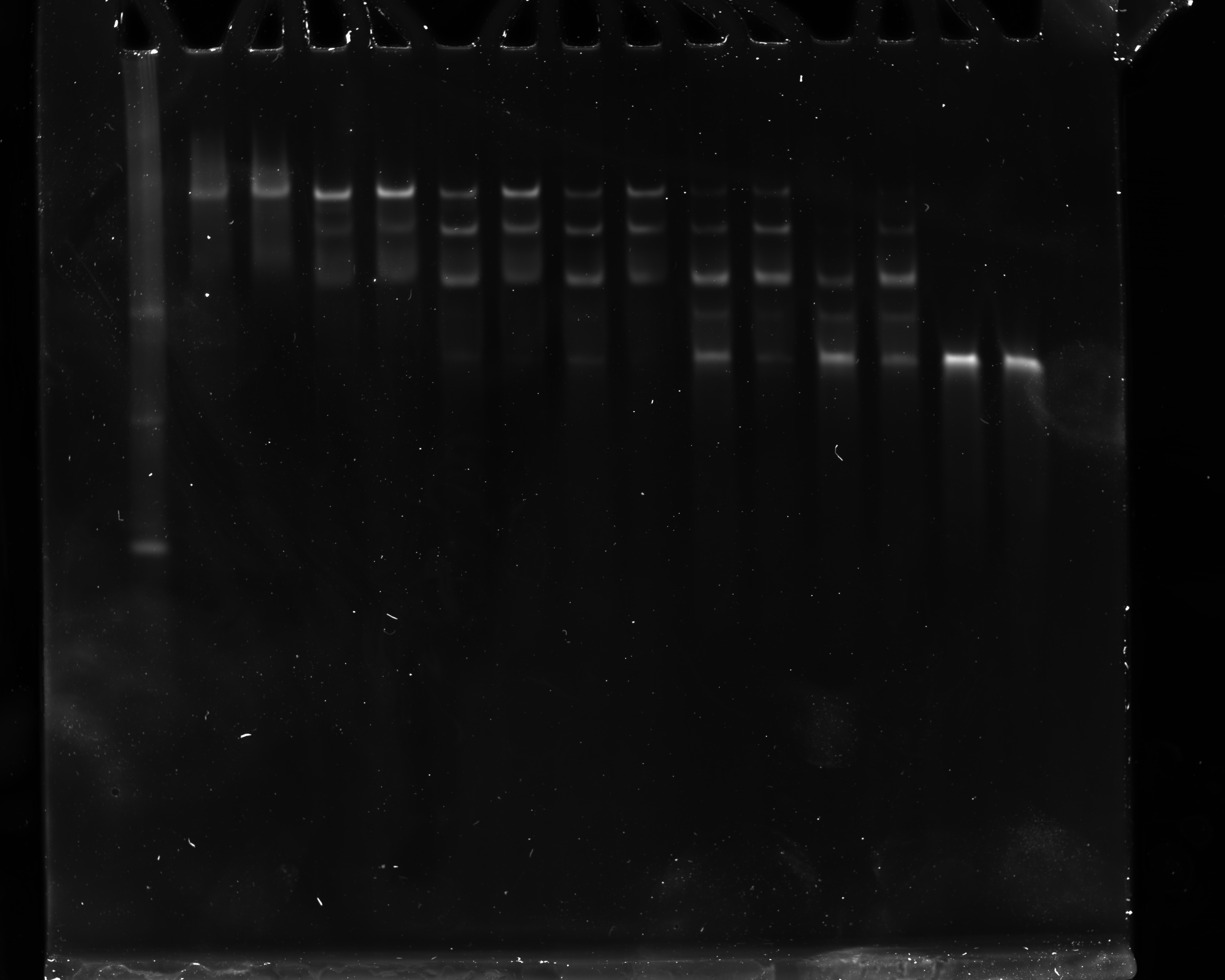

Supplement: Supplementary file 10 — Source Data [file 41467_2024_53400_MOESM10_ESM.zip › Supplementary Figure 2E/SupplementaryTable2_EMSA_EIF2A_WT_Y396E_B.tif]

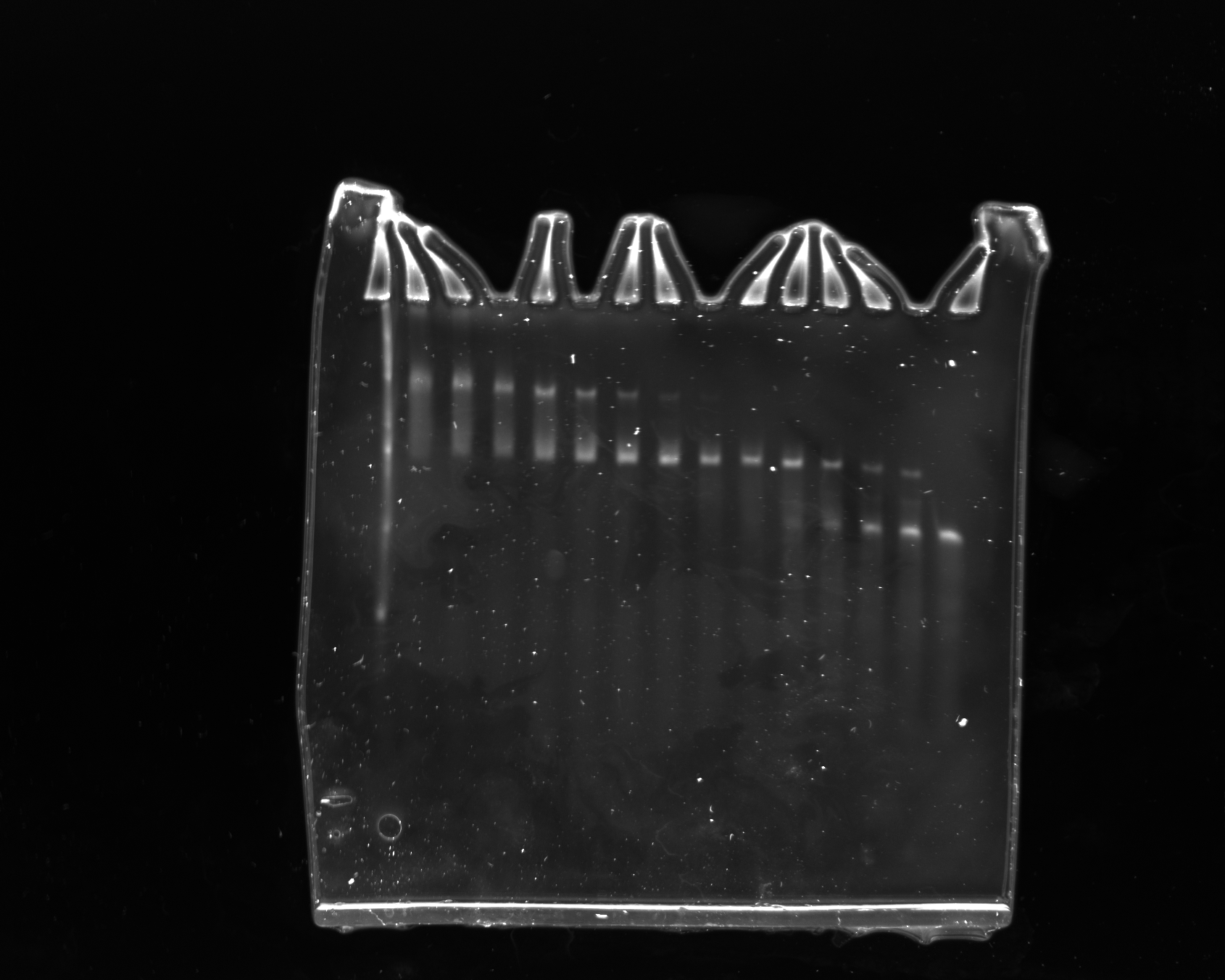

Supplement: Supplementary file 10 — Source Data [file 41467_2024_53400_MOESM10_ESM.zip › Supplementary Figure 2G/SupplementaryFigure2G_SupplementaryTable2_EMSA_MYC_WT_A.tif]

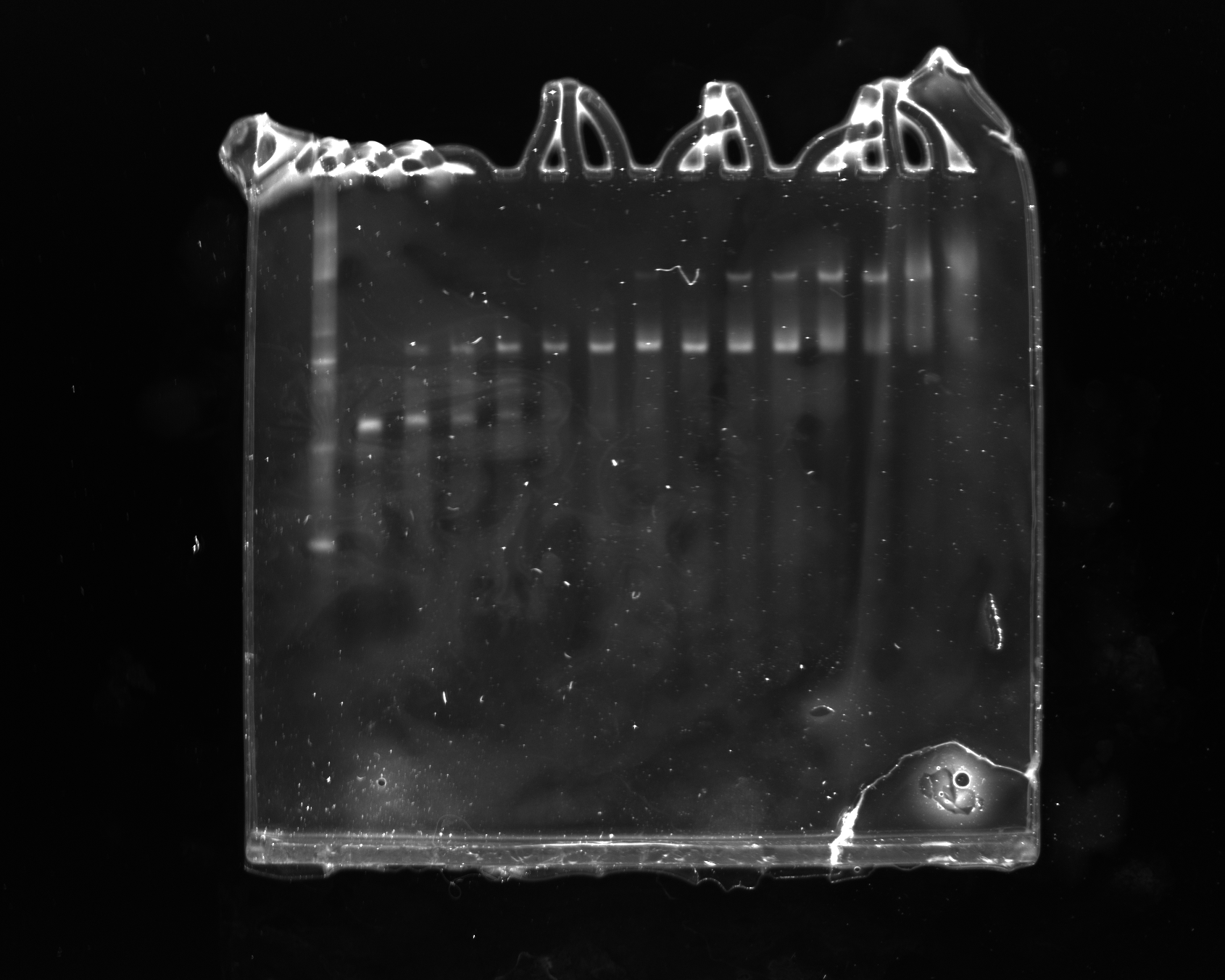

Supplement: Supplementary file 10 — Source Data [file 41467_2024_53400_MOESM10_ESM.zip › Supplementary Figure 2G/SupplementaryTable2_EMSA_MYC_WT_B.tif]

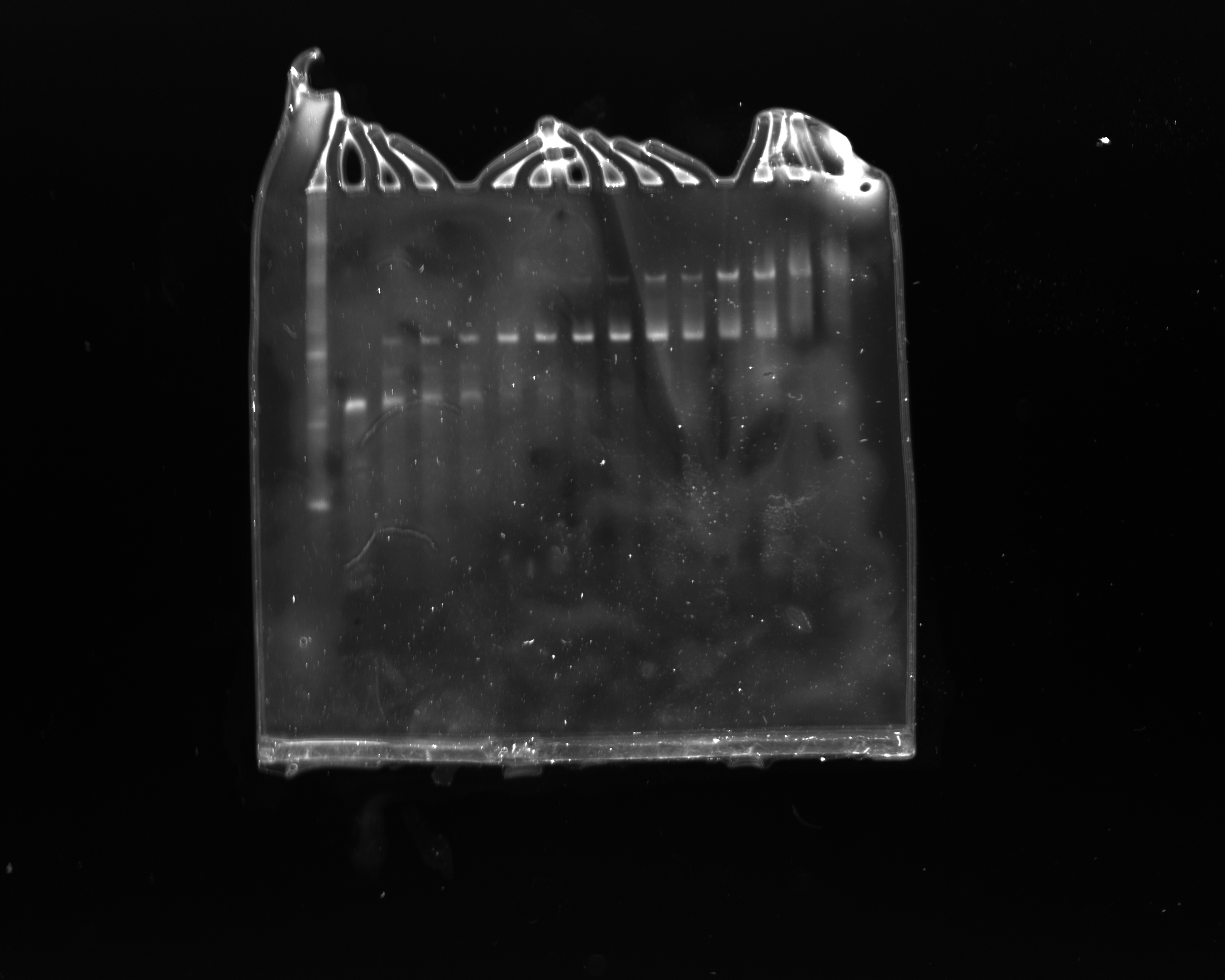

Supplement: Supplementary file 10 — Source Data [file 41467_2024_53400_MOESM10_ESM.zip › Supplementary Figure 2I/SupplementaryFigure2I_SupplementaryTable2_EMSA_MYC_S181E_A.tif]

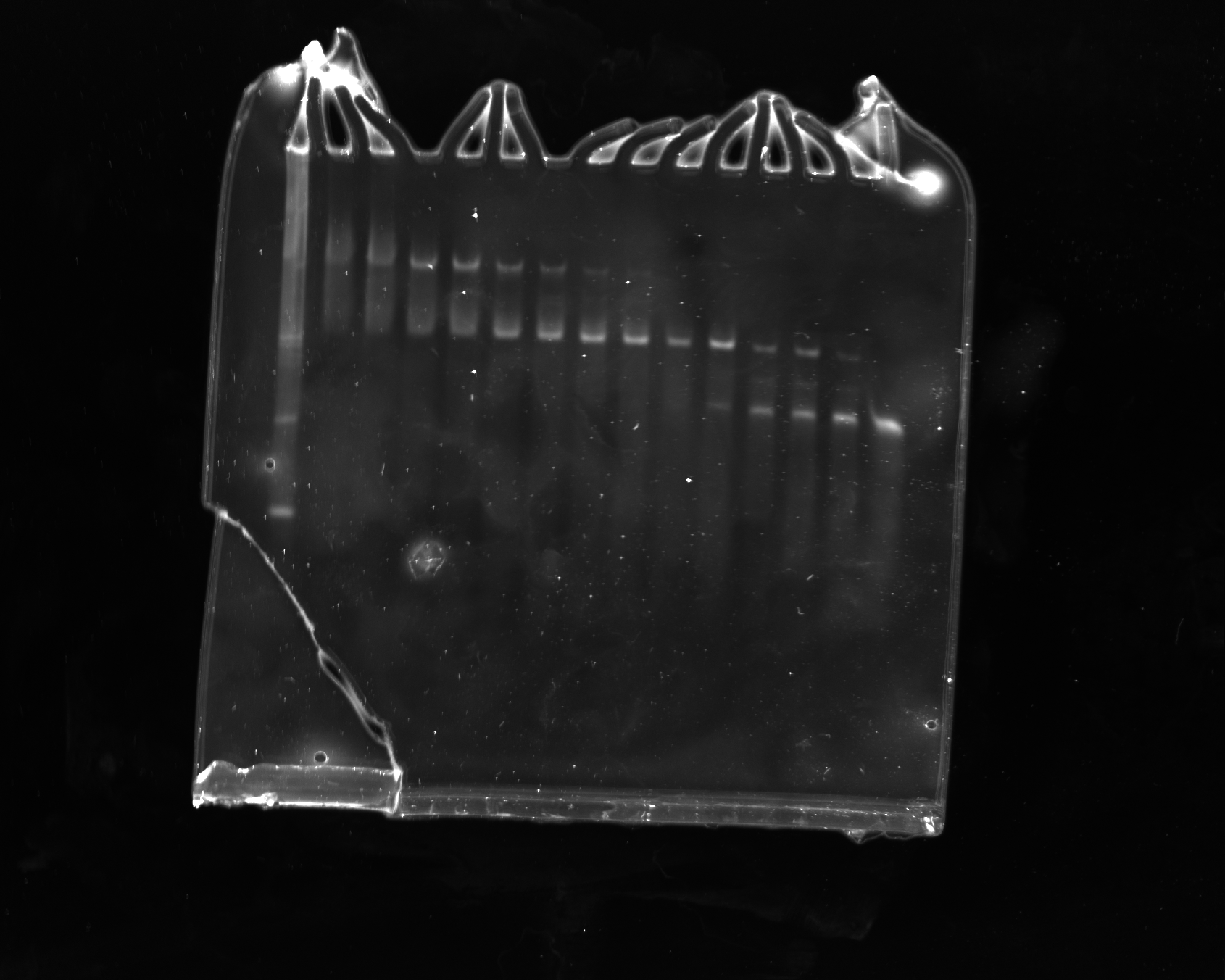

Supplement: Supplementary file 10 — Source Data [file 41467_2024_53400_MOESM10_ESM.zip › Supplementary Figure 2I/SupplementaryTable2_EMSA_MYC_S181E_B.tif]

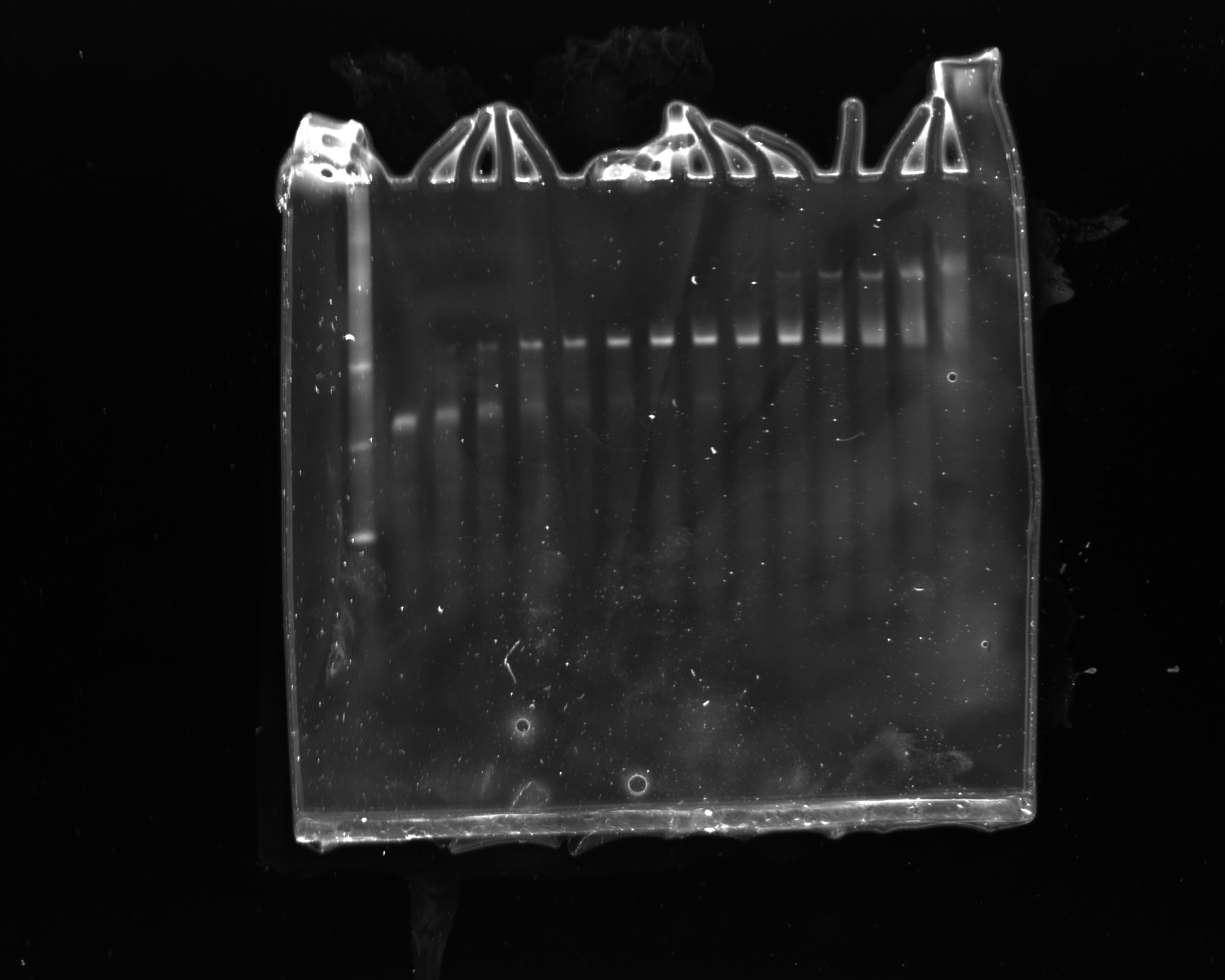

Supplement: Supplementary file 10 — Source Data [file 41467_2024_53400_MOESM10_ESM.zip › Supplementary Figure 2J/SupplementaryFigure2J_SupplementaryTable2_EMSA_MYC_Y396E_A.tif]

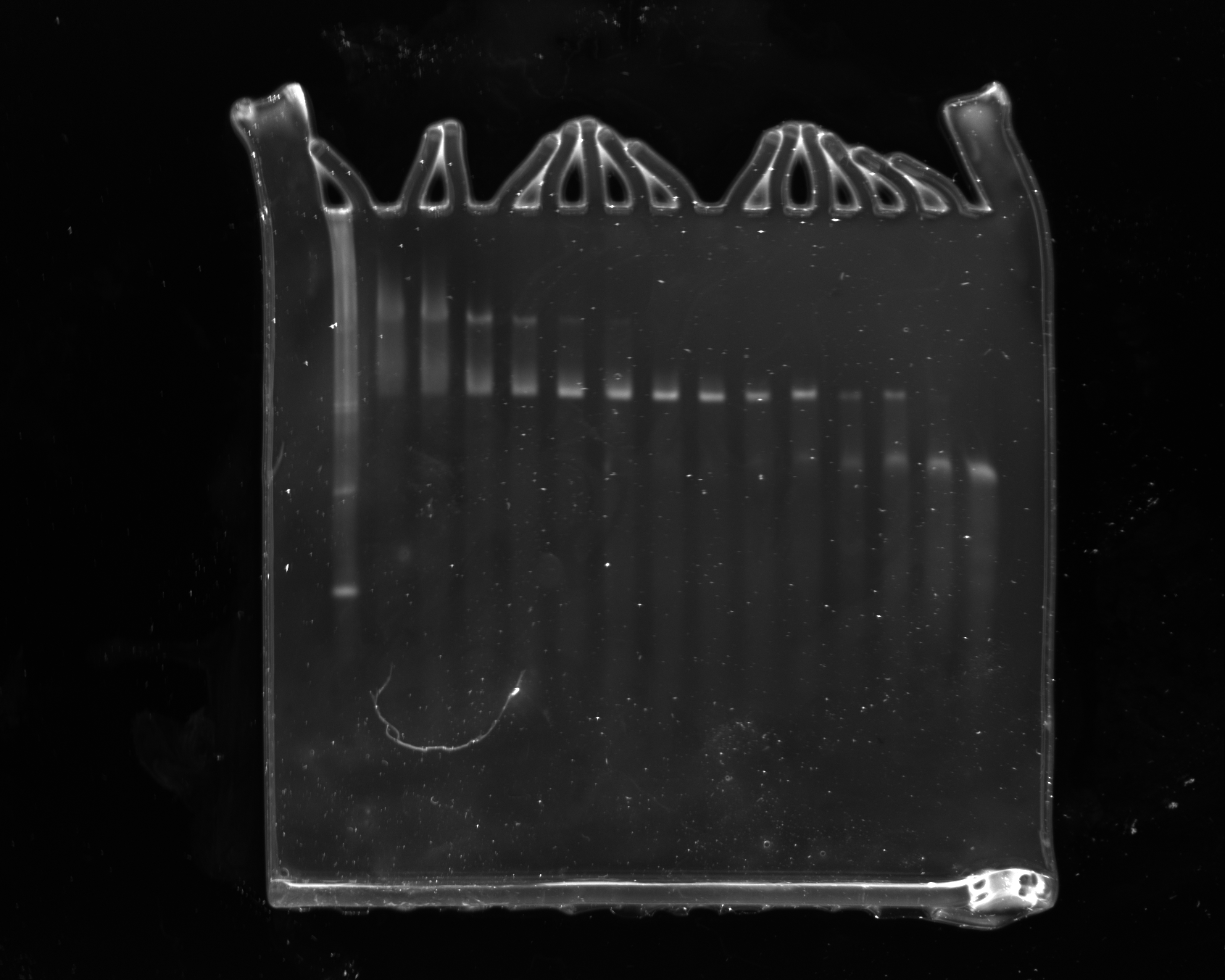

Supplement: Supplementary file 10 — Source Data [file 41467_2024_53400_MOESM10_ESM.zip › Supplementary Figure 2J/SupplementaryTable2_EMSA_MYC_Y396E_B.tif]

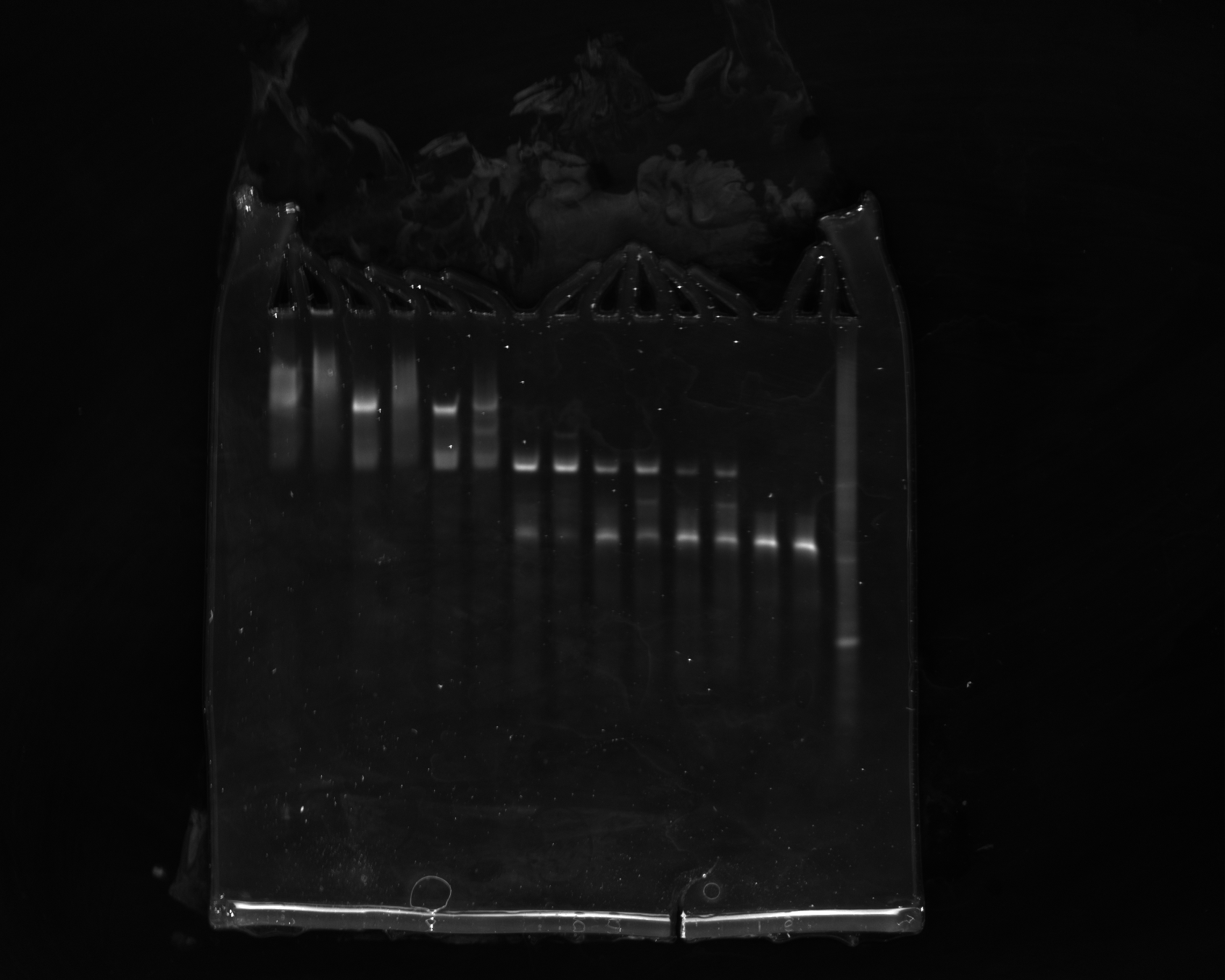

Supplement: Supplementary file 10 — Source Data [file 41467_2024_53400_MOESM10_ESM.zip › Supplementary Figure 3J/SupplementaryFigure3J_SupplementaryTable2_EMSA_XBP1_KH34dR_A.tif]

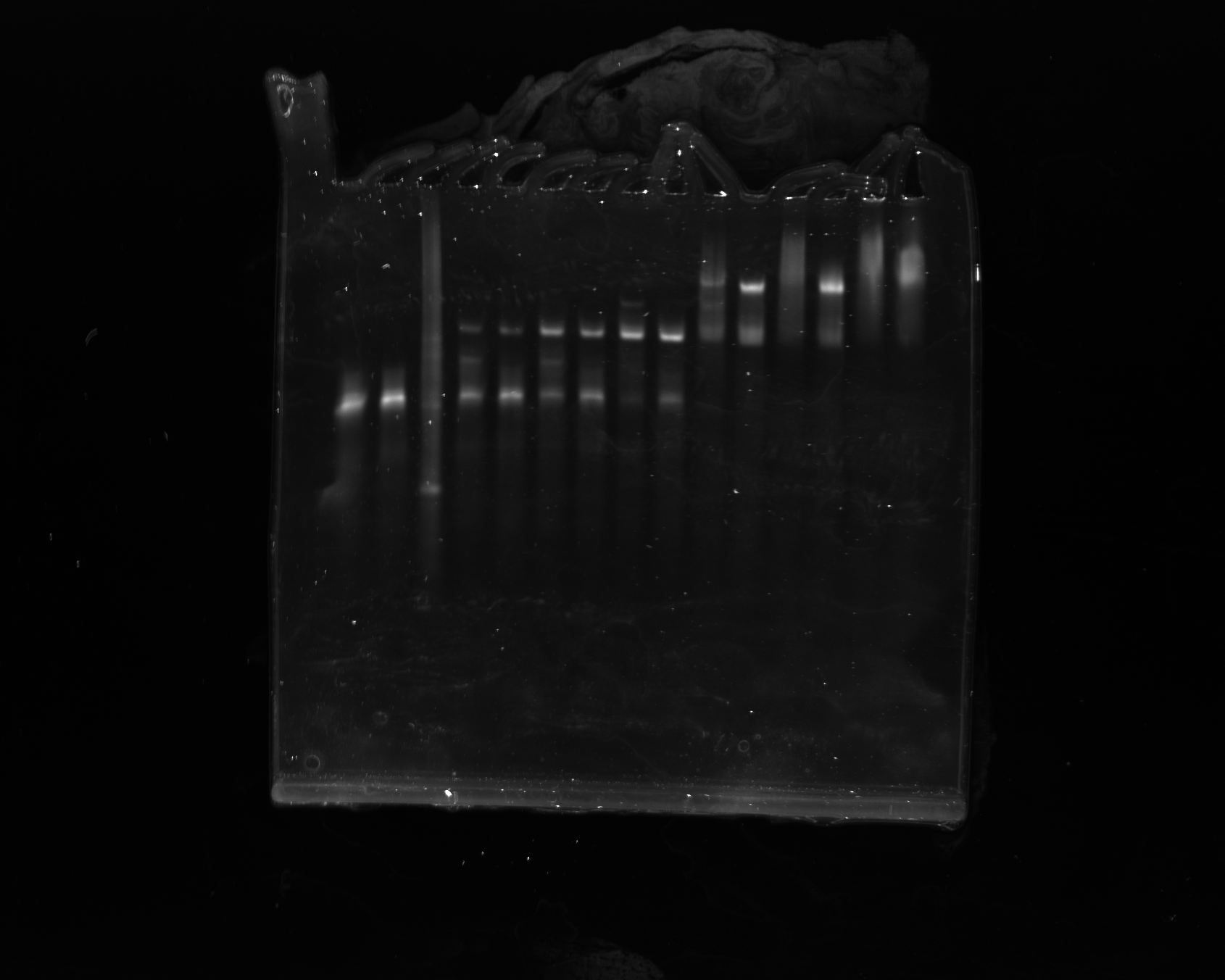

Supplement: Supplementary file 10 — Source Data [file 41467_2024_53400_MOESM10_ESM.zip › Supplementary Figure 3J/SupplementaryTable2_EMSA_XBP1_KH34dR_B.tif]

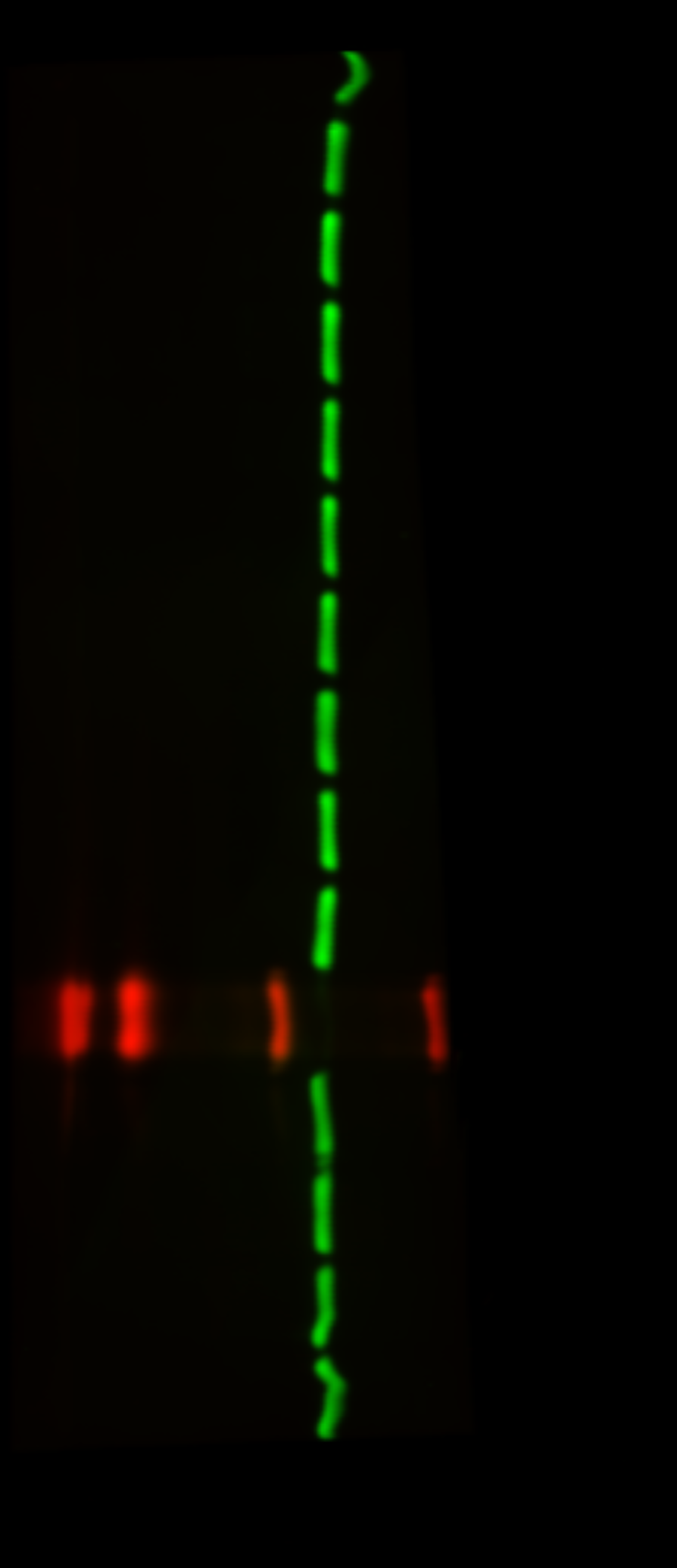

Supplement: Supplementary file 10 — Source Data [file 41467_2024_53400_MOESM10_ESM.zip › Supplementary Figure 6B/SupplementaryFigure6B_HCT116_GAPDH.tif]

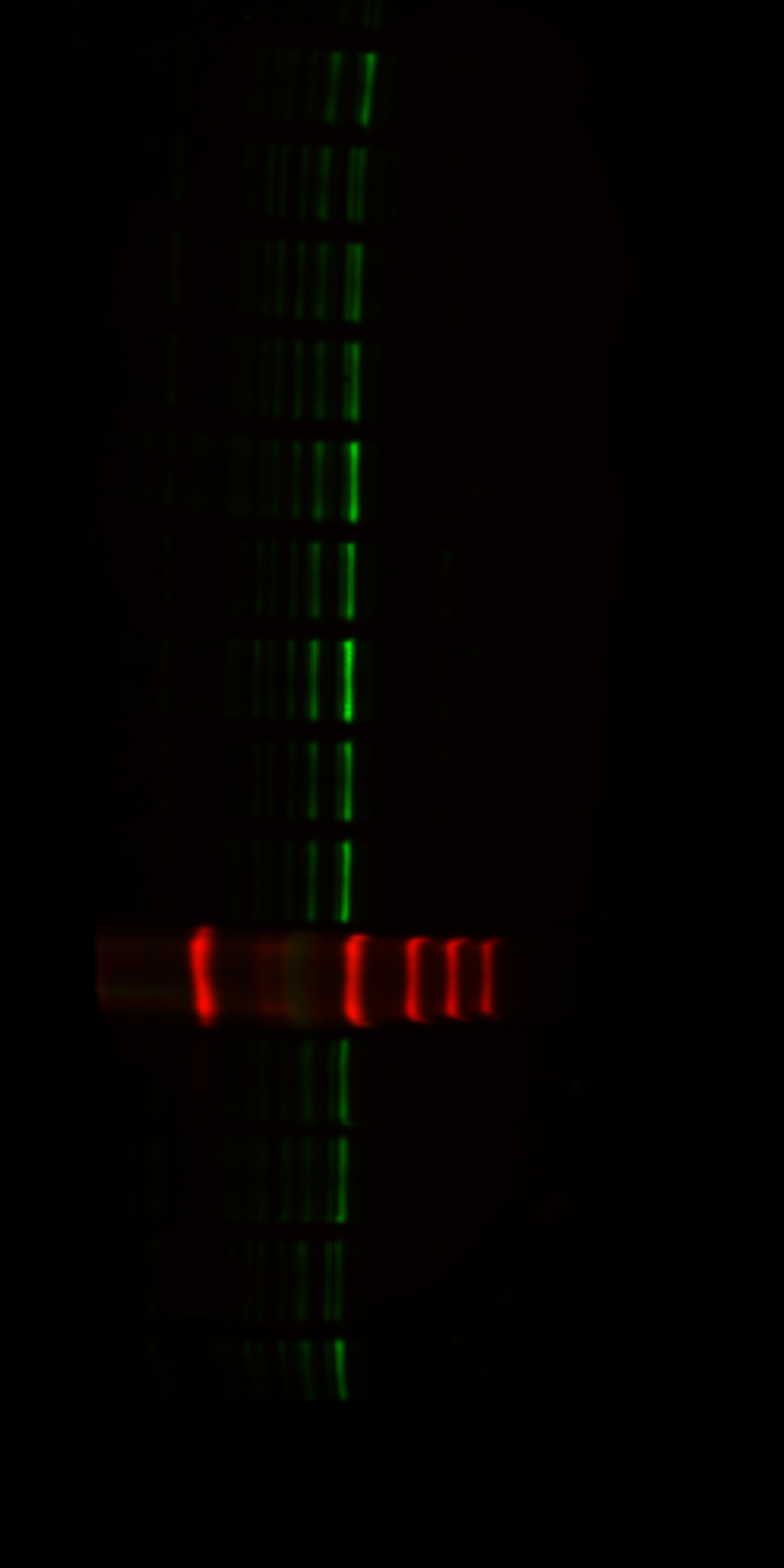

Supplement: Supplementary file 10 — Source Data [file 41467_2024_53400_MOESM10_ESM.zip › Supplementary Figure 6B/SupplementaryFigure6B_HCT116_IGF2BP1.tif]

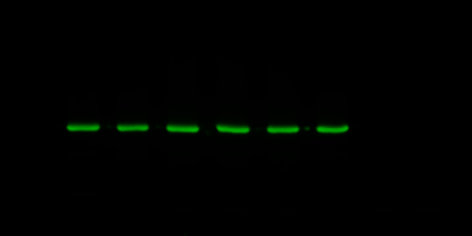

Supplement: Supplementary file 10 — Source Data [file 41467_2024_53400_MOESM10_ESM.zip › Supplementary Figure 6D/SupplementaryFigure6D_U2OS_GAPDH.tif]

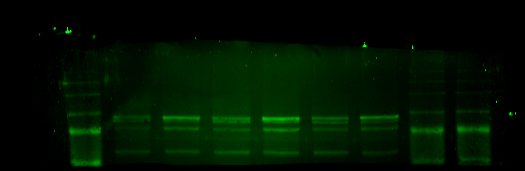

Supplement: Supplementary file 10 — Source Data [file 41467_2024_53400_MOESM10_ESM.zip › Supplementary Figure 6D/SupplementaryFigure6D_U2OS_IGF2BP1.tif]
